# Climate-controlled submarine landslides on the Antarctic continental margin

**DOI:** 10.1038/s41467-023-38240-y

**Published:** 2023-05-18

**Authors:** Jenny A. Gales, Robert M. McKay, Laura De Santis, Michele Rebesco, Jan Sverre Laberg, Amelia E Shevenell, David Harwood, R. Mark Leckie, Denise K. Kulhanek, Maxine King, Molly Patterson, Renata G. Lucchi, Sookwan Kim, Sunghan Kim, Justin Dodd, Julia Seidenstein, Catherine Prunella, Giulia M. Ferrante, IODP Expedition 374 Scientists, Jeanine Ash, François Beny, Imogen M. Browne, Giuseppe Cortese, Laura De Santis, Justin P. Dodd, Oliver M. Esper, Jenny A. Gales, David M. Harwood, Saki Ishino, Benjamin A. Keisling, Sookwan Kim, Sunghan Kim, Denise K. Kulhanek, Jan Sverre Laberg, R. Mark Leckie, Robert M. McKay, Juliane Müller, Molly O. Patterson, Brian W. Romans, Oscar E. Romero, Francesca Sangiorgi, Osamu Seki, Amelia E. Shevenell, Shiv M. Singh, Isabela M. Cordeiro de Sousa, Saiko T. Sugisaki, Tina van de Flierdt, Tim E. van Peer, Whenshen Xiao, Zhifang Xiong

**Affiliations:** 1 School of Biological and Marine Sciences, University of Plymouth, Plymouth, UK; 2 Antarctic Research Centre, Victoria University of Wellington, Wellington, New Zealand; 3 National Institute of Oceanography and Applied Geophysics—OGS, Trieste, Italy; 4 Department of Geosciences, UIT—The Arctic University of Norway, Tromsø, Norway; 5 College of Marine Sciences, University of South Florida, St Petersburg, FL USA; 6 Earth and Atmospheric Sciences, University of Nebraska, Lincoln, USA; 7 Department of Earth, Geographic, and Climate Science, University of Massachusetts, Amherst, MA USA; 8 Institute of Geosciences, Christian-Albrechts-University of Kiel, Kiel, Germany; 9 Department of Earth Sciences, Binghamton University, State University of New York, Binghamton, NY USA; 10 Ocean Climate Response & Ecosystem Research Department, Korea Institute of Ocean Science and Technology, Busan, Republic of Korea; 11 Division of Glacial Environment Research, Korea Polar Research Institute, Incheon, Republic of Korea; 12 Department of Earth, Atmosphere and Environment, Northern Illinois University, DeKalb, IL USA; 13 Florence Bascom Geoscience Center, U.S. Geological Survey, National Center, Reston, VA USA; 14 National Science Foundation, Alexandria, VA USA

**Keywords:** Natural hazards, Geomorphology, Sedimentology, Geophysics, Ocean sciences

## Abstract

Antarctica’s continental margins pose an unknown submarine landslide-generated tsunami risk to Southern Hemisphere populations and infrastructure. Understanding the factors driving slope failure is essential to assessing future geohazards. Here, we present a multidisciplinary study of a major submarine landslide complex along the eastern Ross Sea continental slope (Antarctica) that identifies preconditioning factors and failure mechanisms. Weak layers, identified beneath three submarine landslides, consist of distinct packages of interbedded Miocene- to Pliocene-age diatom oozes and glaciomarine diamicts. The observed lithological differences, which arise from glacial to interglacial variations in biological productivity, ice proximity, and ocean circulation, caused changes in sediment deposition that inherently preconditioned slope failure. These recurrent Antarctic submarine landslides were likely triggered by seismicity associated with glacioisostatic readjustment, leading to failure within the preconditioned weak layers. Ongoing climate warming and ice retreat may increase regional glacioisostatic seismicity, triggering Antarctic submarine landslides.

## Abstract (web summary)

Changes in climate preconditioned large-scale, recurrent Miocene to Pleistocene Antarctic submarine landslides through variations in biological productivity, ice proximity and ocean circulation, posing tsunami risk to Southern Hemisphere populations.

## Introduction

Submarine landslides are global geohazards that can displace huge volumes of sediment, exceeding the size of their terrestrial counterparts by several orders of magnitude^1^. These landslides can generate tsunamis, which may have significant socio-economic consequences through destroying human life, seafloor equipment and infrastructure^2,3^. For example, the 1929 Grand Banks submarine landslide-generated tsunami off Canada generated tsunami waves with a 13 m runup that killed residents along the Newfoundland coast, impacted the coast of Portugal, and caused significant economic damage by severing trans-Atlantic telecommunications cables^4,5^. In 1998, a submarine landslide near Papua New Guinea generated tsunami waves that killed 2200 people^6^. In the mid-Holocene, the Storegga submarine landslide off Norway produced tsunami waves with 20 m runup that likely impacted populations along the southern North Sea coast, Iceland and Greenland, >900 km away^7^. In the southern hemisphere, tsunami waves arriving from South America, New Zealand, and Southeast Asia have been observed around Antarctica and models indicate that centimetre-to-metre scale tsunami waves may impact Antarctica’s margins;^8,9^ thus, tsunami waves originating from Antarctica could reverse this path. Because few submarine landslides are documented around Antarctica^10^, little is known about the probability and potential of southern hemisphere socio-economic impacts of tsunami waves originating from Antarctica’s continental margins. This potential geohazard risk, coupled with increased international interest in subsea internet cable connections to Antarctica, highlights a critical need to improve understanding of downslope processes specific to glaciated continental margins^11^.

Glacially influenced continental shelves make up a fifth of Earth’s continental margin area and are particularly sensitive to climate changes^12^. It is not clear if the low numbers of Quaternary submarine landslides on the Antarctic margin reflect the region’s limited geophysical data coverage or if Antarctica’s slopes are relatively stable due to more homogenous margin sediments, lower regional sedimentation rates, or increased sediment compaction via ice sheets^10^. In contrast, the extensively surveyed northern hemisphere high-latitude margins host some of Earth’s largest Holocene submarine slope failures, which may result from changes in marine sedimentation rates and lithologic properties, gas hydrate dissociation, ice-proximal glacial dynamics, seismicity due to glacioisostatic rebound, and relative sea-level changes^2,13–19^. Because glacial ice transports large volumes of poorly sorted sediment towards the shelf edge, changes in glacial dynamics can influence ice-proximal submarine landslide occurence^16^. Rapid sedimentation decreases pore fluid dissipation, leading to overpressure, under-compaction and a reduction in the sediment effective stress, causing slope failure^17,18^. As many high-latitude submarine landslide triggers (e.g. gas hydrate dissociation, rapid sea level rise, seismicity due to glacioisostatic rebound, and changes in sedimentation^2,13,19^) are sensitive to climate perturbations, it is hypothesised that ongoing and future climate warming may increase the likelihood (or frequency) of high-latitude submarine landslides and associated tsunamis^2,13,19^. Thus, understanding the factors that precondition slopes to fail and mechanisms triggering high-latitude submarine landslides is essential for predicting the timing and location of future slope failures with ongoing climate warming^20^.

On glacial-interglacial timescales, observations indicate that changes in sedimentation on high-latitude margins can result in weak layer formation^21–23^. During interglacials in the circum-Antarctic, diatom productivity and/or sedimentation has been observed to increase within the seasonal sea ice zone while sedimentation rates are typically lower (e.g. few cm kyr^−1^ ^24^) and under the influence of contouritic, plumitic, hemipelagic/pelagic processes^21–23^. Recent studies highlight the importance of climate-influenced diatom production/sedimentation (e.g. diatom oozes) in weak layer formation and submarine landslide occurrence, where climatic changes such as variations in sea-ice cover and ocean temperatures have been shown to affect diatom abundance^23,25,26^. Failure along weak layer planes results from loading and overpressurisation by younger, rapidly deposited glaciogenic or glaciomarine sediments^18,23,26^. Differences in the strength and sediment composition of overlying layers is recognised as a key factor preconditioning slope failure^14,15,23^. However, because of the challenges associated with characterising and constraining weak layers (e.g. limited seismic and geotechnical data resolution, availability and dating uncertainties), failure planes are often difficult to identify^20,23^. Thus, establishing the environmental conditions that predispose slopes to fail on glacial continental margins remains a challenge.

**Fig. 1:**
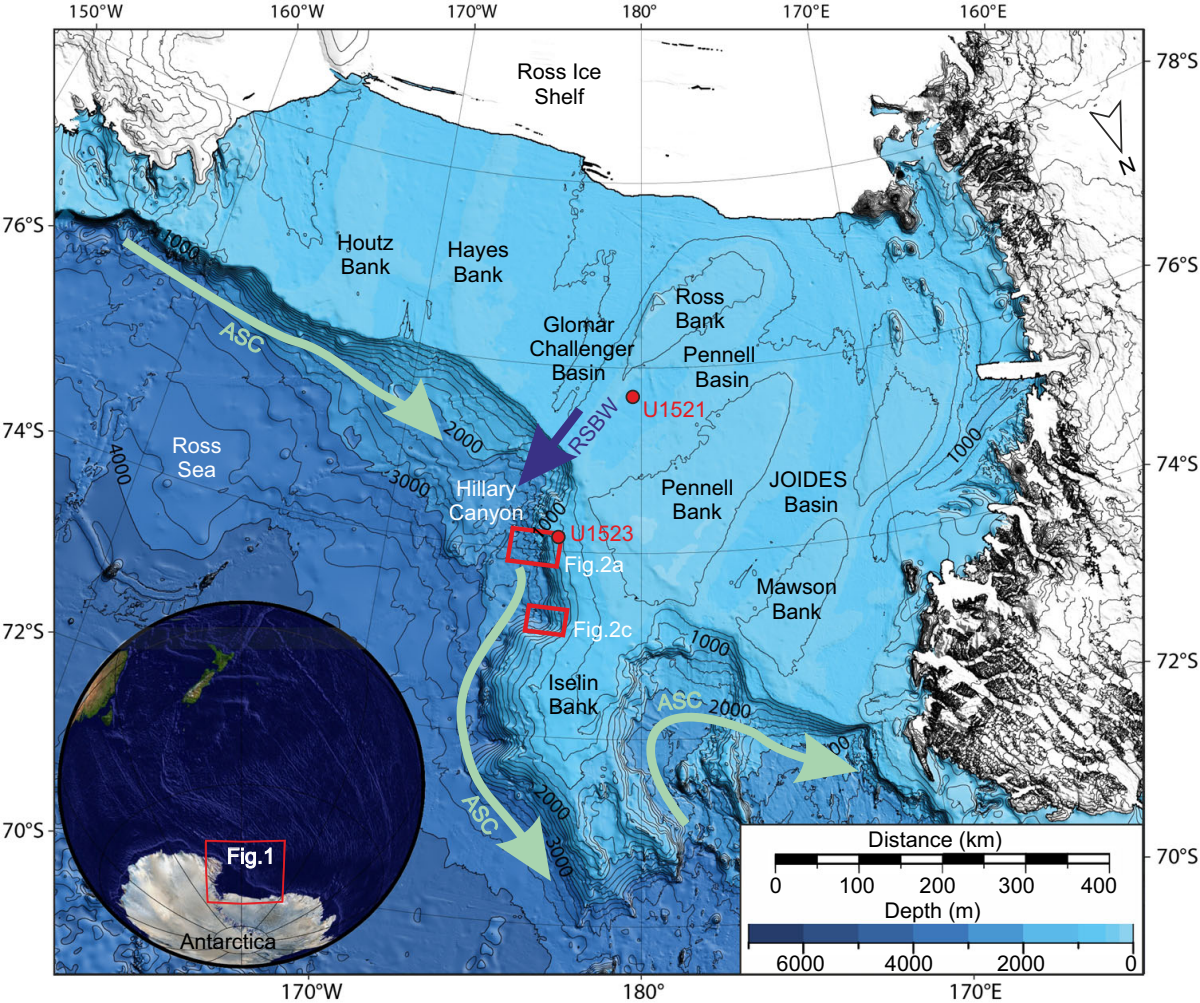
Location of study area on the Ross Sea margin. Inset map locates Fig. 1. in Antarctica. Red boxes show location of Fig. 2a and c. Red circles show locations of IODP Site U1523 and U1521. IBCSO v2 regional bathymetry^90^ with contours spaced at 200 m. ASC is Antarctic Slope Current (shown by light green arrows). RSBW is Ross Sea Bottom Water (shown by dark blue arrow).

The Iselin Bank, located on the eastern Ross Sea continental shelf (Antarctica), adjacent to the southwest-northeast trending Hillary Canyon (Fig. 1), consists of packages of tabular, lens, and wedge-shaped stratified units separated by a series of unconformities above a faulted continental basin^27^. The eastern Ross Sea is part of the West Antarctic Rift System, where extension in West Antarctica likely initiated during the late Cretaceous (~100 Ma) and ceased by the early Miocene^28^. This part of the margin is now considered tectonically passive. The Ross Sea continental shelf is cut by glacially carved troughs eroded by repeated glacial advances since the early Miocene (~18 Ma; Fig. 1)^29–31^. In most of the Ross Sea, grounded ice likely extended to the shelf edge during repeated glacial expansions but not to the Iselin Bank shelf-edge^32^. Grounding zone wedges ~80 km west of the Iselin Bank are probably associated with maximum Last Glacial Maximum ice extent, consistent with model-based reconstructions^33–35^. Contourite mounds observed on the Iselin Bank outer shelf, slope, and continental rise are tens to hundreds of metres thick^36^.

Presently, the Antarctic Slope Current (ASC) flows westward along the continental shelf break and across the Iselin Bank, with average bottom-current velocities of ~0.1–0.3 ms^−1^ (Fig. 1)^36,37^. Warm Circumpolar Deep Water (CDW; *T* > 1.0 °C; *S* < 34.7)^38^ moves onto the continental shelf via bathymetric lows at the shelf edge^39^ and mixes with surface and shelf waters to form modified CDW (mCDW; *T* < −1 °C; *S* < 34.6)^38,40^. Cold dense Ross Sea Bottom Water (RSBW; *T* < −1.85 °C; *S* < 34.7)^40^ forms via brine rejection during sea-ice formation and cools and mixes during sub-ice shelf circulation. Hillary Canyon, adjacent to Iselin Bank, forms a major conduit for RSBW, which flows down the canyon at velocities up to 1 m s^−1^ and mixes with CDW to form Antarctic Bottom Water (*T* = < −1 °C; *S* < 34.6)^38,40^.

Here, we provide a high-resolution (centimetre-to-metre scale), multidisciplinary analysis of Neogene to Quaternary (<23 Ma) submarine landslide preconditioning and triggering on the Antarctic continental margin. We integrate downhole-log data with lithologic, chronologic, and seismic data recovered from the Ross Sea continental margin during International Ocean Discovery Programme (IODP) Expedition 374 to identify multiple weak layers beneath a large submarine landslide complex and provide minimum ages for these submarine landslides obtained by dating stratigraphic horizons immediately overlying submarine landslide scarps. Chronologic data indicate that long-term climatic shifts in the Neogene and Quaternary may have played a critical role in the formation of distinct lithological contrasts that form weak layers prone to failure. Our insights will inform future investigations of geohazards associated with continued climate warming and Antarctic ice retreat.

## Results

### Submarine landslide morphology and stratigraphy

**Fig. 2:**
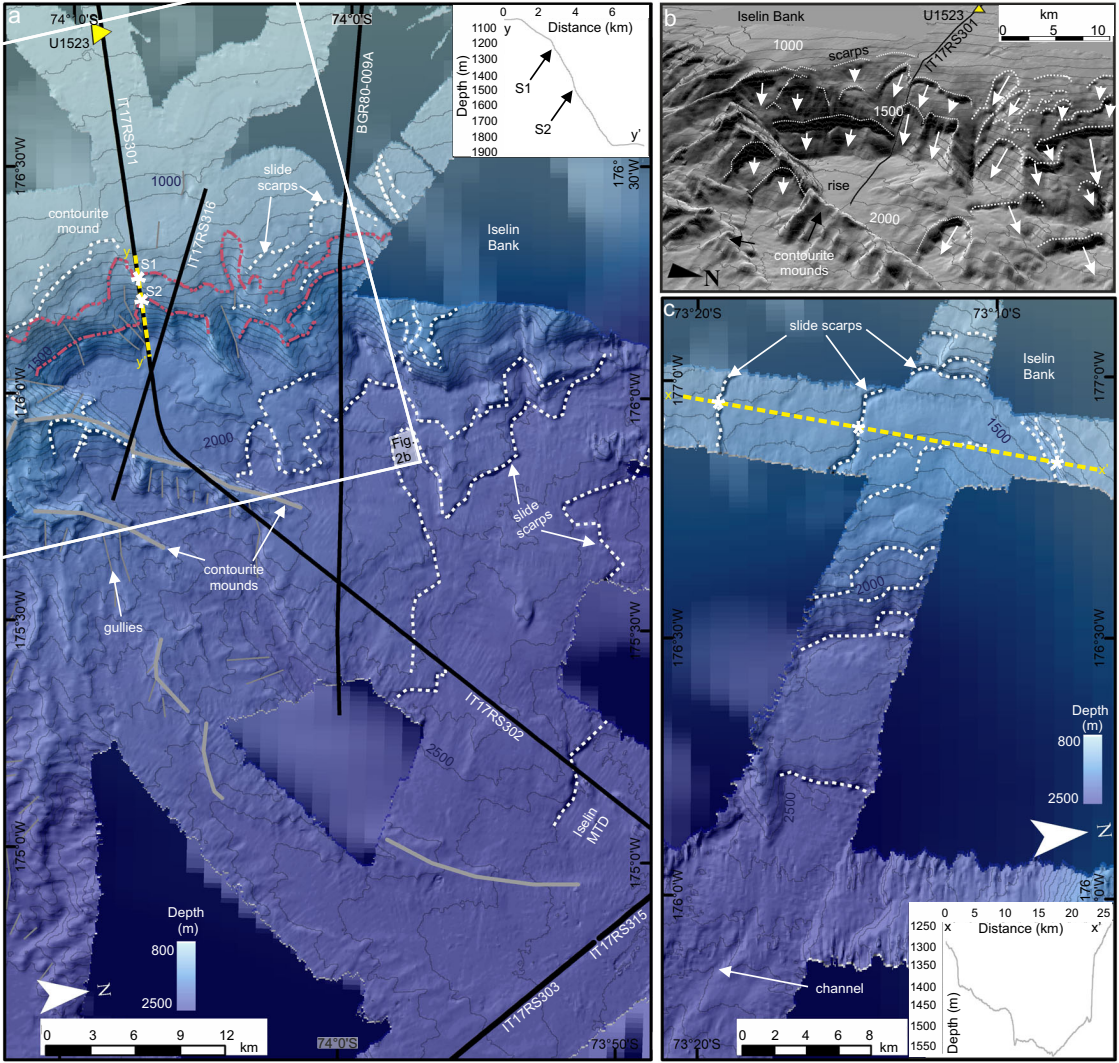
Morphology of the Iselin Bank submarine landslide complex, eastern Ross Sea margin. **a** Southern submarine landslide region. Hillshaded multibeam echosounder data gridded at 30-m cell size overlying IBCSO v2 regional bathymetry^90^. Contours are spaced at 50 m and labelled every 500 m. White dashed lines locate submarine landslide scarps on the slope and rise. Main scarps S1 and S2 are shown by red dashed lines. Thick grey lines indicate contourite mounds’ crest. Black solid lines show locations of seismic lines IT17RS301, IT17RS302, IT17RS303, IR17RS315, IT17RS316 and BGR80-009A. Yellow dashed line indicates location of inset slope profile y-y’ with position of S1 and S2 shown by white asterisks. White box shows location of Fig. 2b. Yellow triangle shows position of IODP Site U1523. MTD is mass-transport deposit. **b** Perspective view of hillshaded multibeam echosounder data gridded at 30-m cell size. Contours are spaced at 50 m and labelled every 500 m. Black line locates seismic profile IT17RS301. Yellow triangle locates IODP Site U1523. White dashed lines show scarp locations. White arrows highlight scarp evacuation paths on the slope and rise. **c** Northern submarine landslide region. Hillshaded multibeam echosounder data gridded at 30-m cell size overlying IBCSO v2 regional bathymetry^90^. Contours are spaced at 50 m and labelled every 500 m. White dashed lines locate submarine landslide scarps on the slope and rise. Yellow dashed line indicates location of inset slope profile x-x’. White asterisks show scarps included in submarine landslide volume calculation.

A large submarine landslide complex of >6000 km^2^ (including evacuation and deposit area; Fig. 2) extends >100 km along the Iselin Bank upper slope (average slope gradients: ~6.5˚). The complex consists of multiple submarine landslide scarps (herein scarps) and headwalls >100 m high and is divided into northern and southern regions based on bathymetric data availability (Fig. 2a  b, c, Supplementary Fig. 1). The seafloor is relatively smooth between the scarps, revealing multiple exposed bedding planes. The southern submarine landslide region consists of two main along-slope scarps (S1 and S2) with volumes of ~19 km^3^ (water depth: 1116 m; area: 141.5 km^2^), and ~13 km^3^ (water depth: 1500 m; area: 106 km^2^; Supplementary Fig. 2). Smaller isolated scarps occur between the main along-slope scarps and four large scarps (in places >100 m high) occur on the continental rise (Fig. 2a). The northern submarine landslide region is characterised by a large, crescent-shaped failure with a volume of >70 km^3^ (water depth: 1300 m; area: >370 km^2^; Fig. 2c). Five large (<180 m) scarps occur on the continental slope (Fig. 2c).

**Fig. 3:**
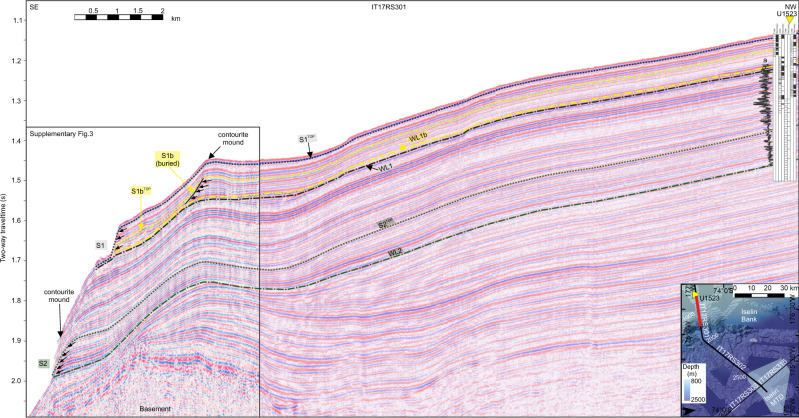
Core-log-seismic integration on the Iselin Bank. Seismic line is IT17RS301 (section). Yellow triangle locates IODP Site U1523 with holes and core recovery shown by black shaded intervals in overlaid core-log. Magnetic susceptibility down-hole log (a) is shown alongside core log. For detail, see Fig. 8. Submarine landslide scarps are labelled S1, S1b and S2. Weak layers (WL1, WL1b, WL2) are located by black dash-dotted line (WL1), yellow dash-dotted line (WL1b) and green dash-dotted line (WL2). Black (S1^Top^), yellow (S1b^Top^) and green (S2^Top^) dotted lines show correlation lines of post-failure reflectors (package immediately overlying the submarine landslide scarp traced into the unfailed slope). Contourite mounds labelled as per Conte et al.^36^. Black box locates NW limit of Fig. 4a and a magnified section is shown in Supplementary Fig. 3. Vertical exaggeration is 12. Inset figure shows hill shaded multibeam echosounder data gridded at 30-m cell size. Contours are spaced at 50 m and labelled every 500 m. Black solid lines show locations of seismic profiles. Yellow triangle locates hole U1523. MTD is mass-transport deposit.

**Fig. 4:**
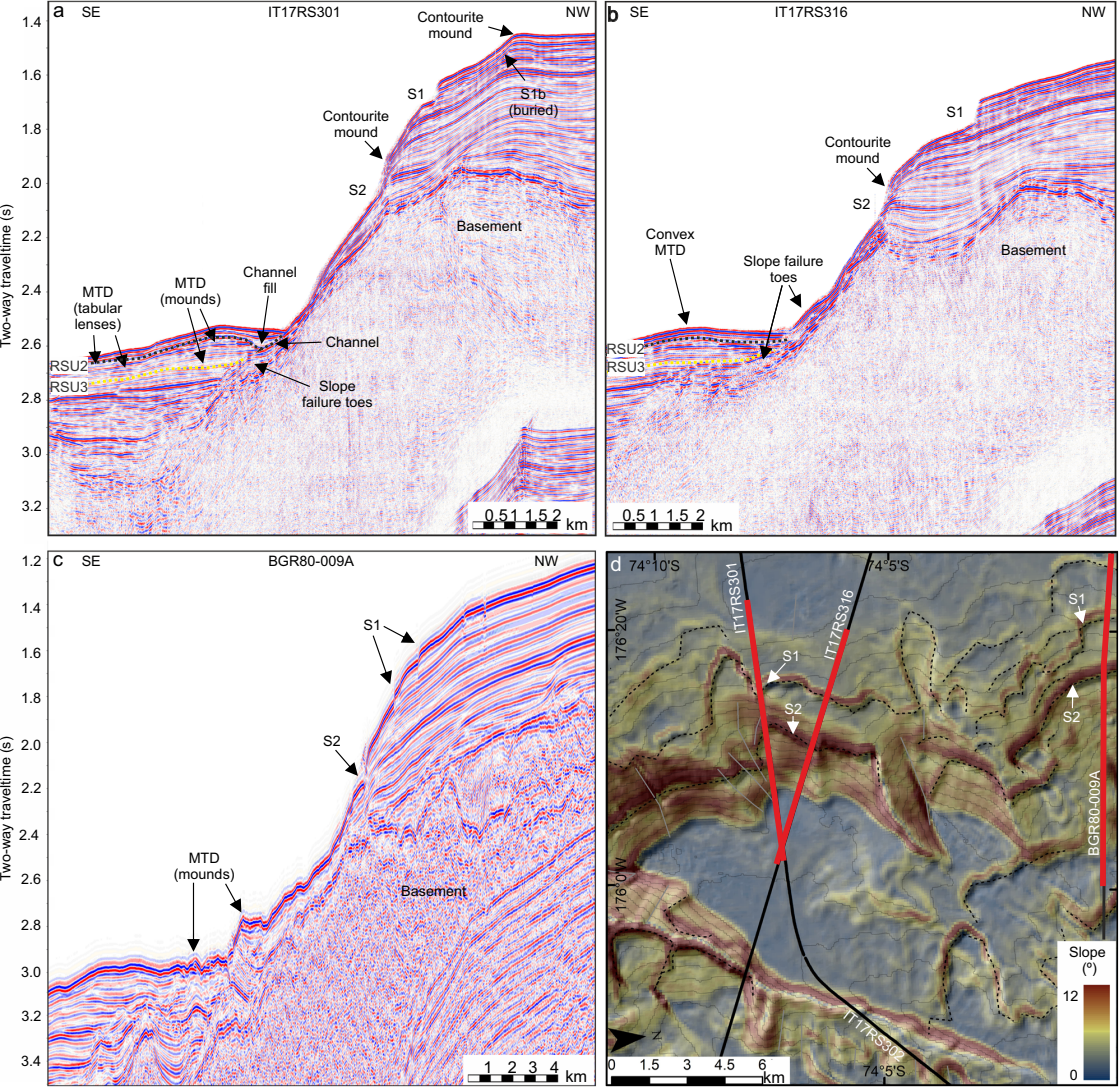
Submarine landslide scarps on the Iselin Bank continental slope. **a** Seismic line IT17RS301 (section). Submarine landslide scarps are labelled S1, S1b and S2. Vertical exaggeration is 8. Contourite mounds as per Conte et al.^36^. **b** Seismic line IT17RS316 (section). Submarine landslide scarps (S1 and S2) are labelled. Vertical exaggeration is 8. **c** Seismic line BGR80-009A (section). Submarine landslide scarps (S1 and S2) are labelled. Vertical exaggeration is 8. **d** Slope gradient (◦) map of Iselin Bank outer shelf, slope and rise. Black solid lines show locations of seismic lines IT17RS301, IT17RS302, IT17RS316 and BGR80-009A. Red lines indicate sections shown in (**a**), (**b**) and (**c**). Submarine landslide scarps (S1 and S2) are labelled. Black dashed lines show extent of submarine landslide scarps.

Seismic lines (IT17RS301, IT17RS316 and BGR80-009A) cross the headwalls of two exposed scarps (S1 and S2) and one buried scarp (S1b) in the southern submarine landslide region^36^ (Figs. 3,  4). Landward of the scarps, the seismic data show stratified, parallel, high-medium amplitude seismic reflectors that can be traced from the shelf edge to IODP Site U1523, located ~15 km south of S1 and S2. The seismic data show that the slope failed along three parallel and continuous bedding planes (Fig. 3), identified as the topmost continuous reflection beneath each scarp, which we refer to as weak layers. Weak Layer 1 (WL1) outcrops at ~1.72 s Two-Way-Time (TWT) at the shelf edge beneath submarine landslide 1 (S1) and is characterised by a medium-amplitude seismic reflection. Weak Layer 1b (WL1b) occurs beneath buried submarine landslide 1b (S1b) and outcrops at ~1.68 s TWT at the shelf edge. Weak Layer 2 (WL2) occurs at ~1.99 s TWT at the shelf edge beneath submarine landslide 2 (S2) and is characterised by a laterally continuous, medium-amplitude seismic reflection overlain by continuous packages of sediment (Fig. 3). WL1/WL1b and WL2 can be traced laterally northward along-slope for >15 km, consistently outcropping beneath S1 and S2 (Fig. 4, Supplementary Fig. 5). Limited resolution of seismic line BGR80-009A inhibits our ability to distinguish WL1 and WL1b along-slope.

**Fig. 5:**
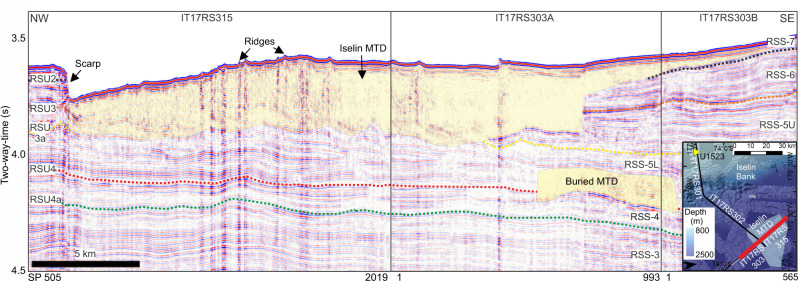
Mass-transport deposits (MTD) on the Ross Sea continental rise. Seismic lines are IT17RS315 and IT17RS303. Vertical exaggeration is 8. Yellow shading highlights extent of MTDs. Ross Sea Unconformities (RSUs) and Ross Sea Sequences (RSSs) derived by Conte et al.^36^. Inset figure shows location of seismic lines (red) on the Ross Sea continental rise. Hillshaded multibeam echosounder data is gridded at 30-m cell size. Contours are spaced 50 m apart and labelled every 500 m. Extent of Iselin MTD is shown as a grey-shaded polygon. Yellow triangle locates IODP Site U1523.

Seismic line IT17RS301 shows a thick chaotic sedimentary unit at the base of the slope, partially buried by stratified parallel subhorizontal reflections and interlayered with opaque tabular lenses and mounds, representing small mass-transport deposits (MTDs; Fig. 4). The MTDs are 1–2 km long and ~25 ms TWT thick (~20 m; see Methods). A channel crosscuts the western flank of the MTD complex at the slope base and is filled with sediments with a chaotic acoustic signal. Seismic lines IT17RS303 and IT17RS315 show two MTDs on the continental rise (Fig. 5). The Iselin MTD is semi-transparent, covers >960 km^2^, has a maximum thickness of ~0.3 s TWT (~240 m) and volume of ~230 km^3^. The buried MTD (~4.1 s TWT), is rectangular in shape, has an area of ~50 km^2^ and is 0.17 s TWT thick (~136 m), indicating a minimum volume of ~7 km^3^.

### Weak layer sedimentology and downhole physical properties

**Fig. 6:**
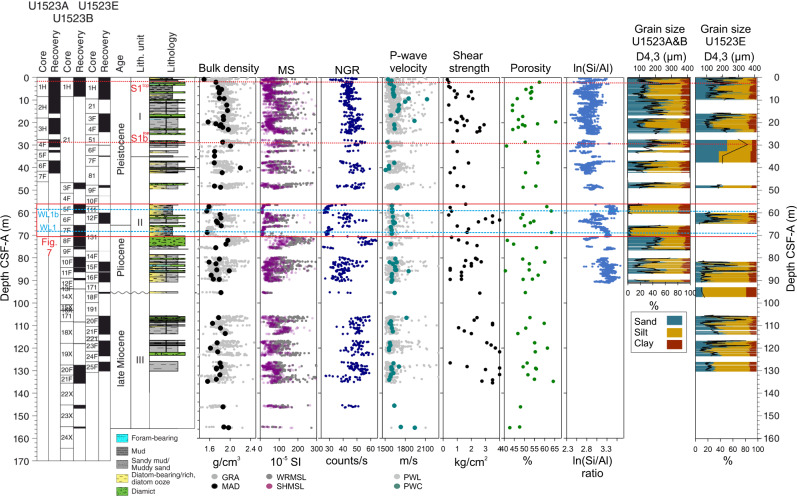
Physical properties, XRF and grain size (fine fraction; 0−1 mm) data from IODP Site U1523. Blue dashed lines show the position of weak layers WL1 and WL1b. Red dashed lines show positions of post-failure reflectors S1^Top^ and S1b^Top^. Red box locates the extent of Fig. 7. Lithology adapted from McKay et al.^35^. MS is magnetic susceptibility; NGR is natural gamma radiation; CSF-A is distance from sea floor to target within recovered core.

**Fig. 7:**
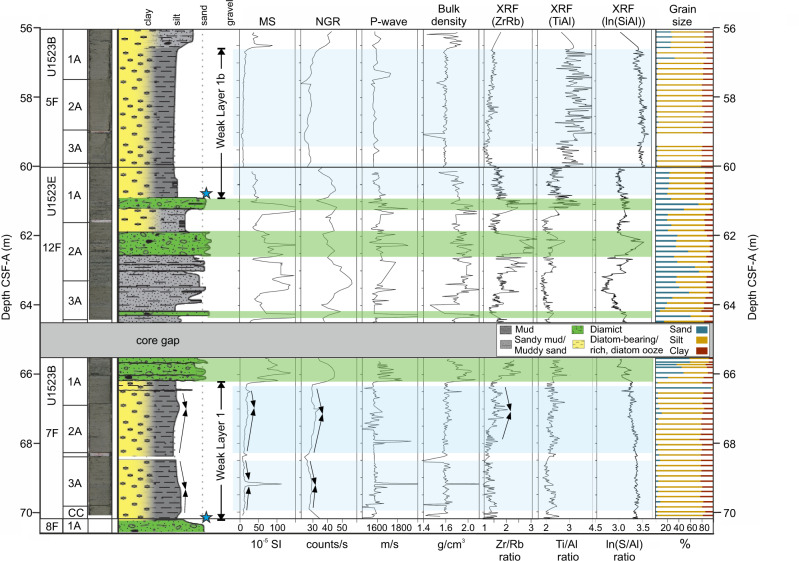
Detailed core log, physical properties, XRF and grain-size data (fine-fraction; 0−1 mm) between 56 and 70 mbsf showing sedimentology of weak layers WL1 and WL1b and overlaying sediment packages. For location in relation to the entire hole, see red box in Fig. 6. Green shading highlights coarser, diamictic sediment packages. Blue shading highlights fine-grained contouritic sediments. Black arrows indicate bigradational grading. Blue stars highlight locations of abundant, well-preserved diatoms. MS is magnetic susceptibility; NGR is natural gamma radiation; XRF is X-Ray Fluorescence; CSF-A is the distance from sea floor to target within recovered core.

WL1 occurs beneath S1 and within core U1523B-7F (66.2–70.2 m below sea floor, mbsf; Figs. 6, 7). WL1 occurs within a ~4-m thick bed of bioturbated muddy diatom ooze with dispersed clasts and multiple millimetre-to-centimetre scale silt and occasional sand stringers throughout. The matrix is characterised by high porosity (~63%) and moderate grain size (D4,3 = 51 μm), magnetic susceptibility (MS; 68 × 10^−5^ SI), bulk density (~1.6 g cm^-3^), and shear strength (1.65 kg cm^−2^). The core scanning XRF-derived element log-ratio of silica to aluminium counts (ln(Si/Al)) shows high values (~3.35) with low Zr/Rb (~1.3) and Ti/Al (~2.1) ratios indicating high sediment opal content. Sediment grain size, XRF, natural gamma radiation (NGR) and MS data indicate bigradational grading with bioturbation and pyrite infilling observed (Fig. 7).

The sediments immediately overlying WL1 are interbedded muds and clast-poor sandy diamicts (Fig. 7; D4,3 = 174 μm). Contacts are sharp to bioturbated with pyrite infilling and glauconite observed throughout. Sand beds tens of centimetres thick (D4,3 = 129.6 μm; ~70% sand) are present, with bioturbated upper and lower contacts. Physical property data show an increase in grain size (D4,3 = 174 μm), bulk density (~2 g cm^−3^), MS (128 × 10^−5^ SI) and shear strength (3.5 kg cm^−2^), and a decrease in porosity (~52%) relative to WL1. Grain size analysis shows that the clay fraction decreases in the overlying package from ~19% (WL1) to ~10% and that the ln(Si/Al) record declines (~3.1), while the Ti/Al (~2.45) and Zr/Rb (~2.1) records increase.

WL1b lies beneath buried S1b and occurs within core U1523B-5F (56.6–60.9 mbsf; Fig. 6; Fig. 7). This interval is characterised by a ~4-m thick bed of bioturbated muddy diatom ooze with dispersed clasts. Physical property data indicate that the beds are very fine grained (D4,3 = 37 μm), with low MS ( ~ 10 × 10^−5^ SI), NGR ( ~ 27 counts s^−1^), bulk density (~1.5 g cm^-3^) and shear strength (0.6 kg cm^−2^) and high porosity (63%). The XRF data reveal high log-ratios of ln(Si/Al). The muddy sand bed overlying WL1b is coarser-grained (D4,3 = 96 μm) and there is a stepwise increase in MS (89 × 10^−5^ SI), NGR ( ~ 40 counts s^−1^), bulk density (~1.9 g cm^−3^), shear strength (1.5 kg cm^−2^), and decreased porosity (61%).

**Fig. 8:**
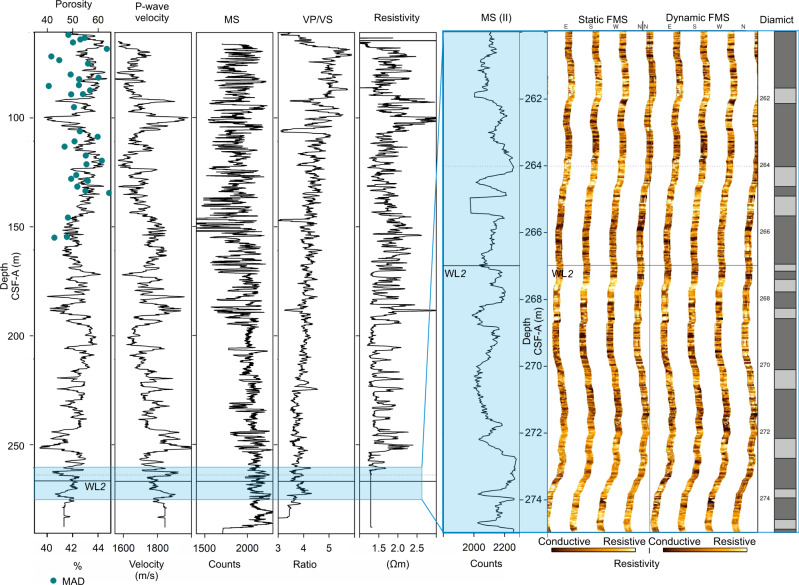
Downhole log and physical property data from Site U1523 shown in relation to core depth scale (CSF-A, m). Blue dots are porosity made by shipboard moisture and density (MAD) measurements (see McKay et al.^35^). MS is magnetic susceptibility. Blue box locates detailed sub-sections shown in MS(II) and Static and Dynamic FMS images. Black horizontal line locates weak layer 2 (WL2). Black dotted line locates package above WL2. Diamict panel shows interpretation of diamictic/gravel-rich intervals (light grey).

WL2 is located at ~267 mbsf and its physical properties are only available from downhole-log data due to extremely low core recovery below ~155 mbsf (Fig. 8). WL2 is characterised by moderate downhole sonic velocity (~1780 m s^−1^) and MS (~2070 counts) as well as moderate-high porosity (~42%). Formation MicroScanner (FMS) resistivity images show a low resistivity interval, with submeter-scale variations in low to medium resistivity, indicating interbedded diatom-bearing to -rich muds. High-resistivity spots indicate occasional clasts. Vp/Vs for WL2 is ~3.96, indicating shaly intervals with elevated porosity (see Methods). The package immediately above WL2 (~264 mbsf; Fig. 8) is characterised by increased downhole sonic velocity (~1880 ms^−1^) and MS (~2260 counts) and decreased porosity (~41%). FMS images indicate this interval has generally high resistivity, with common high resistivity spots indicative of clasts in diamict and/or gravel. The Vp/Vs ratio decreases slightly, indicating decreased porosity.

### Age model and chronology

**Fig. 9:**
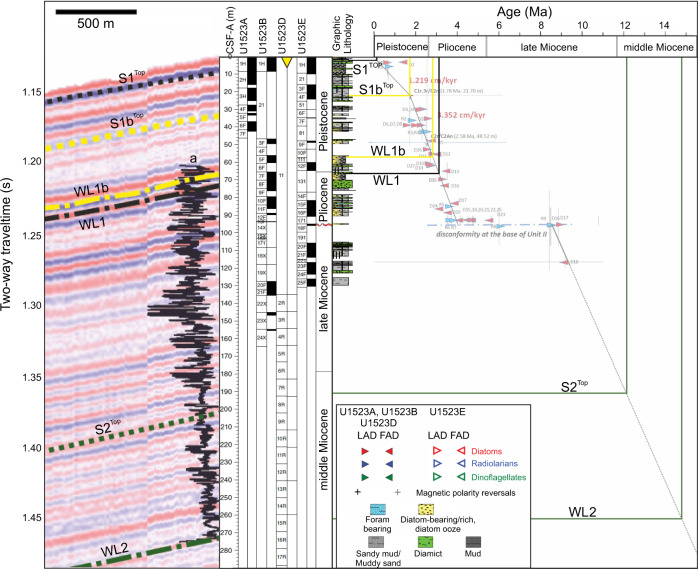
Revised age model for Site U1523 (see Supplementary Fig. 8). Core-log-seismic correlation shown at left. Black line (a) on seismic profile is downhole magnetic susceptibility measurement (see Fig. 8 for detail). Yellow triangle marks Site U1523. Dash-dotted lines on seismic profile locate weak layers: WL1 (black), WL1b (yellow) and WL2 (green). Dotted lines on seismic profile locate minimum submarine landslide ages (post-failure reflector directly above submarine landslide scarp): S1^Top^ (black), S1b^Top^ (yellow) and S2^Top^ (green). Corresponding solid lines on age model show correlation lines for weak layers and minimum submarine landslide ages. For detail, see age model (Supplementary Fig. 8).

We present a revised age model for IODP Site U1523 (Fig. 9, Supplementary Fig. 8; Supplementary Table 1). Core-seismic correlations show that minimum weak layer ages are WL1b: ~ 2.82 Ma (age range 2.93–2.82 Ma) and WL1: ~ 3.07 Ma (age range 3.21−3.07 Ma; see Methods). Within the WL1b and WL1 intervals (56.6–60.9 and 66.2–70.2 mbsf, respectively) there are unique samples containing very rich, well-preserved diatoms with rare planktic foraminifera (Fig. 7). The richest diatom samples contain only traces of reworked diatoms from older ages and represent primary pelagic biogenic sedimentation. WL2 is below the maximum depth of the age model (220 mbsf), but is likely older than ~13 Ma, based on the last appearance datum (LAD) of diatom *Denticulopsis lauta* (~13.0 Ma) and first appearance datum (FAD) of diatom *Nitzschia denticuloides* (13.5 Ma) in the deepest diatom bearing sample at 221.55 mbsf^35^. If the sedimentation rate remained constant and the age model linearly extrapolated to the WL2 depth of 267 mbsf, this surface would correlate to ~14.8 Ma. However, the validity of this assumption remains equivocal due to limited recovery (52% below 120 mbsf; Fig. 9)^36^. Minimum submarine landslide ages, based on core-seismic correlation of the post-failure reflectors (horizons directly above each of the submarine landslide scarps: S1^Top^, S1b^Top^ and S2^Top^; Fig. 3) traced into the adjacent unfailed slope to Site U1523, are S1: < 400 ka, S1b: ~1.72 Ma, and S2: ~12.14 Ma (see Methods; Fig. 9).

Regional core-seismic integrations show that the buried MTDs in the distal sector of the continental rise (Fig. 5) incise Antarctic Regional Seismic Unconformity (RSU)-4 and are overlain by seismic Ross Sea Sequence (RSS)−5L and RSU3a^36,41^, indicating an age range of ~11–14.5 Ma. The Iselin MTD incises RSS-7 and RSU2 to RSU3a, suggesting an age <2.5 Ma^36^. Small MTDs younger than RSU3 (3–5 Ma)^36^ occur at the Iselin Bank slope base (Fig. 4).

## Discussion

Interpretations of core-downhole log-seismic and chronological data from the eastern Ross Sea have implications for constraining submarine landslide preconditioning and triggering around Antarctica. Large-scale recurrent slope failures have occurred along >100 km of the Iselin Bank since at least the middle Miocene, with weak layers identified beneath three submarine landslide scarps. We propose that global climatic changes influenced weak layer formation on the Ross Sea shelf and slope by creating distinct lithological contrasts between the diatomaceous weak layers and overlying diamicts that preconditioned slopes to fail. This study indicates the importance of climate in preconditioning slope failure and provides insight into geohazards associated with future warming and ice retreat.

The lithologies and physical properties of weak layers (WL1 and WL1b) reveal thick packages of muddy diatom ooze and diatom-rich mud (Fig. 7), with downhole-log properties indicating WL2 is likely formed of similar hemipelagic, higher porosity, diatomaceous sediments. Increased diatom content affects the geotechnical properties of sediments through increases in water content, opal content, compressibility, permeability, and the angle of friction within sediments^23,25,42–45^. Excess pore pressure can be generated by trapping water and/or quickly decreasing the pore space available due to tectonic stress, compaction, cementation, rapid sedimentation and low permeability of overlying glacial sediments, sometimes promoting slope instability in overlying sediments^23,25,45^.

Sedimentation rates associated with WL1 and WL1b deposition are relatively low (~3.4 cm kyr^−1^; Fig. 9). Modelling studies show that excess pore pressure generated under similar conditions (<15 cm kyr^-1^ ^46^) in comparable fine-grained hemipelagic muds is also low, with conditions unlikely to result in slope failure without an external trigger^18,46,47^. Numerical modelling shows that sediment compressibility is a critical factor in the stability of low gradient slopes^46^, where compressibility increases with increasing biogenic opal content^48^. Diatom oozes and diatom-rich muds, such as those observed in the Iselin Bank weak layers, are more compressible than siliciclastic muds; therefore the process of compaction could release excess fluid that might accumulate if bounded by a lower-permeability unit, leading to weak layer formation^18,22,23,42,47^. Excess pore pressure can also be generated by crushing of in-situ microfossils present in the hemipelagic sediments during compaction^49^, and the expulsion of intraparticle waters stored in their tests^50^. Water released during diagenetic alteration of biogenic silica may also result in slope failure, although this process typically occurs between 300 and 800 mbsf^25^, below the depth of observed Iselin Bank weak layers (between 60 and 270 mbsf) and the opal CT transition zone at IODP continental shelf Site U1521 (~285 mbsf)^35^.

High-density diamicts deposited above low-density diatom ooze and diatom-rich muds likely create lower permeability boundaries that prevent upward migration of pore fluids, leading to increased pore pressure and decreased shearing resistance. Although direct permeability experiments were not conducted, the permeability of the overlying glacigenic packages is likely low, based on porosity-permeability relationships of similar sediments^14,15,45^.

Based on our revised age model for Site U1523, we suggest that abundant, well-preserved diatoms and fine-grained siliciclastic sediments that characterise diatom oozes and diatom-rich muds of regional weak layers (Fig. 7), were deposited at the seasonal sea ice edge or in seasonally ice-free open marine conditions during prolonged intervals of warmer-than-present climates. At present, in circum-Antarctic shelf and slope sediments, high diatom productivity and deposition are associated with seasonally open marine conditions, and often with warmer sea surface temperatures^51^. Core-log-seismic integration reveals ages of ~2.93−2.82 Ma for WL1b, ~ 3.21–3.07 Ma for WL1 and ~14.8 Ma for WL2. These periods correspond with notably warm global climates of the late Pliocene interglacials (e.g. Marine Isotope Stage G11: ~2.83 Ma, and/or G17: ~ 2.95 Ma), the mid Pliocene Warm Period (mPWP: 3.264–3.025 Ma) and the Miocene Climate Optimum (MCO: 17–14.5 Ma) where there were reduced Antarctic ice volumes, higher sea levels, warm Southern Ocean surface waters and regional open marine conditions^52–56^.

Analysis of the planktic foraminiferal assemblages and planktic foraminiferal and diatom geochemistry of Site U1523 provides further evidence of reduced ice volume and warmer-than-present ocean temperatures at the site when WL1b and WL1 were deposited (between ~3.07 and 2.82 Ma)^35,57,58^. Within the WL1b interval (~2.93–2.82 Ma), subtropical and temperate planktic foraminifera dating to ~2.9 Ma indicate distinct warm-water incursions into the Ross Sea^57^. WL1 (~3.07 Ma) corresponds to the mPWP (3.264–3.025 Ma), an interval when mean temperature was 2–4 °C warmer than today^59^ and numerical modelling shows higher sea levels (~25 m higher than today) and reduced Antarctic ice-sheet extent^60,61^. These weak layer intervals correspond with negative foraminiferal and biogenic silica oxygen isotope data^58,62^, and peaks in the XRF data (ln(Si/Al)), a proxy for sedimentary biogenic opal content^63^ are coincident with deep-sea stable isotope data and models which suggest that climate was warmer and ice volume was reduced during WL1 and WL1b diatom ooze deposition^59–61,64,65^. Ice-proximal evidence from ANDRILL AND-1B in the southwestern Ross Sea indicates reduced summer sea-ice extent during peak interglacials prior to 2.58 Ma, and a significant interval of meltwater-rich ice retreat near the G17 interglacial (~2.95 Ma)^53,60^. Continental shelf deglaciation and warming may have resulted in enhanced productivity and greater accumulation of diatom ooze relative to periods of increased summer sea-ice duration before 2.6 Ma^53^.

WL1b and WL1 are overlain by packages of sandy muds, gravels, and diamict (Fig. 7)^35^. The interpretation of gravel-rich strata above WL1b is supported by the inference of hard layers at ~46 mbsf indicated by drilling parameters^35^. This increase in grain size likely reflects a shift towards enhanced sediment delivery from expanded ice sheets and iceberg rafting, combined with enhanced winnowing by invigorated along-slope bottom currents as global and regional climates cooled^36^. Evidence from AND-1B sediments show glacial-interglacial grounding-line oscillations between 3.3 Ma and 2.0 Ma that are associated with stepwise cooling from 2.8 Ma to 2.6 Ma, a cooling trend also observed in deep oxygen records^53^. A significant cooling event occurred at 2.6 Ma, with increased polynya activity, sea-ice presence, and grounding-line advance leading to reduced diatom ooze deposition^53^.

WL2 (~14.8 Ma) is characterised by downhole-log data suggesting the presence of diatom oozes and diatom-rich muds were deposited under seasonally open marine conditions with minimal terrigenous sediment input^66^. This interval of enhanced diatom deposition occurred at the end of the MCO when Southern Ocean temperatures were the warmest of the Neogene and ice volume was reduced^35,54,55,67–69^. MCO-age records from IODP Site U1521 and Deep Sea Drilling Project Site 273 recovered metres-thick sections of diatom oozes and diatom-rich muds, providing additional support for our interpretation of the Site U1523 downhole-log data^35,67^.

The package overlying WL2 is characterised by a diamict-dominated sequence with ice rafted debris (IRD), suggesting an ice-proximal to glacimarine depositional environment. As seismic, lithologic and geomorphological data from the Iselin Bank suggest that grounded ice did not override Site U1523 during full glacial conditions^35^, this change in sedimentation reflects increased bottom-current activity leaving behind coarser-grained lag deposits consisting of IRD, glacimarine outwash, and mass-wasting deposits^35^. The sedimentary package corresponds to RSS-5, above RSU-4 (~14.5 Ma), based on regional seismic correlation^36,68^. Contourite mound growth and slope progradation were also enhanced from ~14.5 Ma to 8 Ma, suggesting that increased sediment discharge, down-slope flows, and bottom-current activity occurred as Antarctic ice sheets expanded and climate cooled^30,36^. This observation is consistent with western Ross Sea evidence for a major discontinuity at ~14.6 Ma coincident with a 30-m decrease in eustatic sea level and cooling of Southern Ocean bottom water temperatures^55,66,70^.

Submarine landslide scarps are associated with a distinct change in the orientation of Iselin bank to a more north-south orientation and where regional oceanographic modelling predicts maximum modern-day ASC velocities^36^ (Supplementary Fig. 9). Modern velocities (>0.2 m s^−1^) are fast enough to deposit bedload and transport fine-grained suspended load^71^. A decrease in average current intensity (to ~0.03 m s^−1^) occurs over the outer shelf to continental rise, leading to sediment deposition and construction of contourite mounds^35,36^ (Supplementary Fig. 9).

Presently, ASC strength and position varies in response to climate changes^37,72^, leading to changes in sediment erosion and deposition, which likely influenced repeated slope failure on the Iselin Bank. By analogy with modern warming, the Neogene interglacials—when weak layer sediments were deposited – were likely associated with easterly winds over the continental shelf, weaker bottom-current strength, and warmer intermediate water access to the shelf, contributing to ice retreat^36,60^. Sediments within WL1b and WL1 are predominantly cohesive, with mud contents between ~95 and 98%, suggesting low bottom-current velocities, which may have resulted from a shift in the position of the core of a proto-ASC current^37^. Increased nutrients at the sea-ice edge likely enhanced diatom productivity and deposition, resulting in diatomaceous sediment deposition in this low energy setting.

In sediment packages overlying the weak layers, changes in sediment type, shown in the elemental composition (XRF), physical properties and downhole-log data, may reflect a northward shift in the pathway and strength of regional bottom currents and the sea-ice edge due to a more northerly position of the core of easterly winds during glacials^72^. Increased bottom-current strength prevents the deposition of (or winnows) finer grained material, consistent with observed grain size increases above the weak layers. As ice did not ground at the Iselin Bank shelf edge, increased terrigenous material in sediments overlying the weak layers likely reflects a combination of changes in the proximity of the ice margin, increased availability and transport of IRD and sediment-laden meltwater^35^. These environmental conditions likely reduced diatom productivity at the shelf break^53,73^.

On the Iselin Bank, slope gradients are enhanced by locally mounded contourite geometries resulting from bottom current-driven sediment deposition (Fig. 4)^36^. This may have caused over-steepening that facilitated slope failure and undercutting at the base of the slope by current scouring, as indicated by the channel observed at the continental slope base, where the present-day ASC has maximum velocity (Fig. 4a, Supplementary Fig. 9). Contourite mounds are associated with slope failure in a range of global settings^74,75^ and can increase pore pressure via high sedimentation rates and rapid loading, as well as sediment characteristics including high-water content, low permeability, and high porosity, leading to under consolidation and gas-associated fluidification^74–77^. Such conditions may have influenced mass wasting along similar high-latitude contouritic settings (e.g. Storegga submarine landslide)^14,78,79^. Columnar blankings observed on Ross Sea continental rise seismic profiles may indicate gas-associated fluid escape^36^. However, no columnar blanking is observed on the shelf or slope surrounding the Iselin Bank scarps. Further, there is no evidence of methane gas or gas hydrates in regional seismic profiles, suggesting that gas hydrate dissociation was unlikely to have triggered slope failure^80^. Even if gas hydrates were present in this area in the past, they were likely stable at the Iselin Bank submarine landslides depths^81^.

Modelling studies of similar sediments and sedimentation rates show that excess pore pressure alone is unlikely to result in slope failure^15,18,45,47^. Thus an external trigger was likely required to destabilise Iselin Bank sediments. Submarine landslide ages on the Iselin Bank may be correlated with broad-scale climate events: S1: < 400 kyr, S1b: ~1.72 Ma, and S2: ~ 12.14 Ma (see Methods for uncertainties). This is based on unique chronologic ties at 21.7 mbsf (C1r.3r/2n: 1.78 Ma) and 48.52 mbsf (C2r/C2An: 2.58 Ma) and FAD and LAD events (see Methods). Direct correlations between the submarine landslides and MTDs on the continental rise are limited by seismic correlation down-slope of U1523. However, regional core-seismic correlations^36,41^ show that S1/S1b may correlate with the Iselin MTD (<2.5 Ma), and S2 with the buried MTD (~11–14.5 Ma)^36^.

**Fig. 10:**
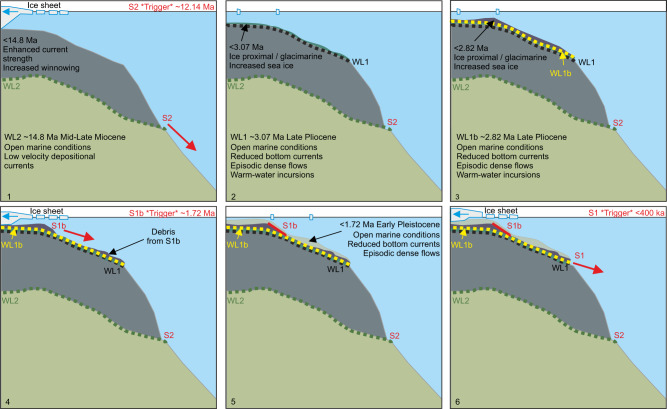
Schematic of submarine landslide and weak layer occurrence on the Iselin Bank from the mid- to Late Miocene (1) to the Late Pleistocene (6). Weak layers are shown by black (WL1), yellow (WL1b) and green (WL2) dashed lines. Submarine landslides are S1, S1b and S2. Red arrows indicate scarp evacuation paths. Solid red line shows location of buried S1b. Panels (1) to (6) show environmental conditions and timing of weak layer and submarine landslide occurrence from the mid- to Late Miocene (1) to the Late Pleistocene (6).

We propose that submarine landslide triggers on the Iselin Bank may be associated with changes in sedimentation following periods of climatic deterioration and/or rapid local uplift following glacial retreat, glacio-isostatic readjustment, and unloading^19,82^. S1b and S2 follow the Plio-Pleistocene transition (3.2–2.6 Ma)^53^ and middle Miocene Climate Transition (~14 Ma)^54,66^. Direct evidence of major ice sheet advances across the Ross Sea continental shelf at these times are unequivocally provided by drill sites on the continental shelf^60,67,69^, followed by periods of extensive ice sheet retreat as climate rebounded in the late Miocene and early Pleistocene^31,83^. The S1 event postdates the Mid-Pleistocene Transition (~1–0.8 Ma; Fig. 9; Supplementary Table 1)^83^ where evidence from ANDRILL cores suggest Antarctic ice sheet grounding lines retreated into the inner continental shelf during Ross Sea interglacials between 400 kyr and present day, compared to more expansive ice sheet coverage between 800 and 400 kyr^83^. We suggest that climatic changes preconditioned slope instability and indirectly influenced submarine landslide occurrence (Fig. 10). This follows previous studies that show no correlation of global submarine landslide age with significant changes in global climate^20^. During periods of climatic warmth (e.g. MCO and mid Pliocene), low density diatom-rich weak layers are deposited. During periods of climatic cooling (e.g. middle to late Miocene and Plio-Pleistocene glacials), more proximal grounded ice increases erosion and terrigenous sediment supply to the outer shelf. On Iselin Bank, diamicts deposited above diatom-rich weak layers preconditioned its slope to fail. Glacial retreat, glacio-isostatic readjustment and unloading may also have led to rapid local uplift^19,82^, increasing earthquake frequency^19^ that could trigger slope failure via liquefaction of weak layers when bounded by lower permeability layers^4,78,84^.

Our study has global implications for large-scale mass-wasting events, as biogenic opal deposition is prevalent in the Southern Ocean and circum-Pacific upwelling zones. However, Iselin Bank’s unique environmental setting may explain the region’s abundance of submarine landslides. Here, continuous along-slope sediment deposition of relatively uncompacted diatomaceous sediments occurs alternating with mud, sand, and gravel-rich sediments. Bathymetric, seismic and core data show that scarps occur at similar depths along-slope and are likely coeval, with weak layers extending >15 km along-slope beneath the scarps (Supplementary Fig. 5, Supplementary Fig. 7). Further, unlike many Antarctic margins, the bathymetry of Iselin Bank shelf edge may prevent iceberg keel scouring and ice-sheet grounding, even during sea level lowstands, resulting in the accumulation and preservation of the observed contouritic deposits. Ice was instead likely frequently grounded at least near to the shelf edge around much of Antarctica^33,65^, preventing the build-up, and subsequent failure, of weak sedimentary packages over multiple glacial cycles, resulting in over-compacted, lithologically uniform and overall stable depositional sequences. The conditions that led to recurrent failure on the Iselin Bank are likely to be present in other high-latitude margins, suggesting that these margins are likely unstable and may pose a significant hazard risk^43^. Our results emphasise the need for future research aimed at understanding the extent and presence of weak layers around Antarctic margins and highlight increased risk of slope instability with ongoing and future rapid warming and ice retreat.

Our study also has implications for predicting submarine landslide frequency and occurrence, the potential for submarine cable breaks, and global tsunami risks. Tsunami wave characteristics are largely controlled by submarine landslide volume, slope gradient, and submergence depth^3^. On the Iselin Bank, submarine landslide volumes exceed 70 km^3^ with MTDs on the continental rise exceeding 230 km^3^. The volumes of sediment displaced are great enough to generate tsunami waves, based on analogous submarine landslides in similar high-latitude depositional settings^5,85,86^. For example, numerical modelling of submarine landslides (10–100 km^3^) on the North Scotia Ridge generated tsunami waves ranging from 1-40 m amplitude^85^. Similarly, the Grand Banks submarine landslide (185 km^3^) caused 3–8 m wave amplitudes^4,5^ and the Storfjorden submarine landslide, Norway (40 km^3^) caused 1–2.5 m wave amplitudes^86^. Recent studies demonstrate teleconnections between Antarctica and Southern Hemisphere landmasses, with centimetre-to-meter scale tsunami waves reaching Antarctica within hours of generation^8,9^. These teleconnections show that tsunami waves originating from Antarctica have the potential to reverse these pathways.

Recurrent slope failure occurred along >100 km of the Iselin Bank slope during the Neogene. Core-log-seismic data show that three submarine landslides occurred above recognisable weak layers characterised by distinct packages of water-rich diatom oozes overlain by dense, water-poor coarse-grained diamicts. Sedimentological and chronologic analyses indicate that past warm climate events, including the MCO and the mPWP, led to enhanced diatom deposition. The weak layers are overlain by low permeability glacigenic sediments likely deposited by down-slope transport of glacimarine outwash, debris-rich icebergs, and winnowing by along-slope currents. We propose that the combination of undisturbed packages of weak, water-rich sediments overlain by dense coarser grained glacigenic sediments increased pore pressure in the biogenic weak layers making the slope susceptible to failure. While the ages of the weak layers correspond to warm climate intervals, the ages of the submarine landslides are not correlated to specific climate events. The trigger required to destabilise the slope was most likely seismicity generated by glacial loading or unloading during periods of rapid marine-based ice-sheet retreat. This study has important implications for understanding the influence of global climate changes on factors influencing slope failure. Given increasing interest in deploying submarine cable networks to Antarctica, similar morphological settings should be prioritised for study elsewhere around Antarctica’s margins to better understand the causes and consequences of large submarine landslides. This is especially pertinent given predictions of future rapid global warming.

## Methods

### Geophysical data

Multibeam echosounder (MBES) and high-resolution single-channel seismic data were acquired during the 2017 ANTSSS and ODYSSEA expeditions on RV OGS *Explora*. Bathymetric data were acquired using a hull-mounted 12 kHz Reason SeaBat 7150 and 8111 MBES and were processed using PDS2000 and Caris Hips and Sips v8.0 to a 30-m cell size. Sound-velocity data were acquired from two sound velocity probe stations. ArcGIS v10.7 was used to create a slope-gradient map (◦) with a 30-m cell size from the bathymetric data. Single-channel seismic-reflection lines IT17RS301, IT17RS302, IT17RS303, IT17RS316 and IT17RS315 were acquired using a linear array of two 210 cu.in GI guns spaced 2 m apart, towed at 4 m water depth, and shot in harmonic mode. The vertical resolution is ~3 m based on a frequency of 130 Hz and sound velocity of 1600 ms^−1^ ^36^. A shot point interval of 8 s was used, corresponding to a shot distance of 13–15 m. A 9.5 mini-streamer receiver array was towed 35 m from the source, which included 10 hydrophones spaced 0.625–1 m apart. Processing and interpretation were done using Schlumberger Vista and Petrel software and included bandpass filtering, deconvolution, horizontal stacking of the 10 traces to produce a single channel configuration, amplitude gain normalisation and time-migration.

Multi-channel seismic reflection line BGR80-009A was acquired by RV OGS *Explora* in 1980 which used a 2400-m, 58-trace streamer and 23.4 l, 24-gun source array. Seismic reflection line BGR80-009A was reprocessed for this study using the open-source Seismic Unix® software and Echos/Geodepth (Paradigm®) software. Processing included static corrections, band-pass filtering, geometrical spreading correction, automatic gain control, horizontal stacking and time migration. The vertical resolution is ~17 m based on a frequency of 23 Hz and sound velocity of 1600 m s^−1^.

Multi-channel seismic reflection line IT94A127A was acquired by RV OGS *Explora* in 1993–94 using a 3000-m, 120 trace streamer and a 74.8 l, 2 × 20-gun source array. The seismic line was reprocessed for this study using the open-source Seismic Unix® software and Echos/Geodepth (Paradigm®) software, with processing including resampling in time and space, and band-pass filtering. Seismic line IT94A127A has a vertical resolution of ~13 m based on a frequency of 30 Hz and sound velocity of 1600 m s^−1^.

A sound velocity of 1600 m s^−1^ was used to calculate the dimensions of seismic features in metres, including MTDs on the continental rise (not crossing site U1523)^36^.

### Submarine landslide volume calculations

Submarine landslide volumes were calculated by creating a digital elevation model representing the smooth pre-submarine landslide surface for each submarine landslide scarp (Supplementary Fig. 2) using ArcGIS v10.7. Interpolation (Topo to Raster tool) was used to estimate the pre-submarine landslide surface by using depth values directly adjacent to the failed submarine landslide headwall (Supplementary Fig. 2). The Cut Fill tool was used to deduct the modern-day bathymetry from the interpolated pre-submarine landslide surface, providing an estimation of the volume difference between the two surfaces. A grid cell size of 30-m was used for the interpolated and modern bathymetry. This calculation provides a minimum submarine landslide volume as data is limited down-slope of the submarine landslide headwalls in places. Only volume difference in regions where modern-day bathymetry is present were calculated.

### Sediment data

Site U1523 was drilled in 828 m water depth on the outermost Ross Sea continental shelf during IODP Expedition 374^35^. The site includes five holes (U1523A-E) drilled in close proximity^35^. Stratigraphic correlation and the composite depth scale incorporates Holes A, B and E and is based on high-resolution (cm-scale) core scanning X-ray fluorescence (XRF) analysis^87^. High-resolution (cm-scale) shipboard physical property measurements were made on the recovered core material^35^. Measurements included: gamma-ray attenuation bulk density (2.5 cm resolution), MS (2.5 cm resolution), compressional (P-wave) velocity (2.5 cm resolution), spectral gamma ray (2.5 cm resolution) and colour reflectance and colorimetry (2.5 cm resolution). Undrained shear strength measurements were made using a handheld Torvane. Moisture and density analyses measured wet and dry bulk density, porosity, and grain density on discrete ~10 cm^3^ sediment samples every ~75 cm down-core. Post-expedition core scanning XRF measurements were made using the Avaatech XRF fluorescence core scanner at the Gulf Coast Repository (1–2 cm sampling resolution). Elemental ratios Zr/Rb, Ti/Al and ln(Si/Al) were used as proxies to show variations in grain-size and sediment opal content^63,88^.

Grain size of the <1 mm fraction was measured every 15 cm down-core using a Malvern Mastersizer 2000 laser particle size-analyser. Each 5 mm^3^ subsample was dry sieved using a 1-mm mesh sieve, a 10% H_2_O_2_ solution was added to remove organic material and the samples were left overnight in a water bath at 60 °C. A 10% solution of sodium hexametaphosphate was added and ultrasound applied before measurement. To analyse the coarse fraction (>1 mm), subsamples of 1 cm^3^ were manually dry-sieved in a nested stack at half-phi intervals from 1.4–16 mm. Grain-size data were analysed using GRADISTAT software according to the following grain-size intervals: very fine-coarse sand (1 mm–62.5 μm), silt (62.5–4 μm) and clay (<4 μm). The volume mean grain-size diameter is presented (D4,3). These data alongside post expedition core descriptions have been used to refine the high-resolution stratigraphic columns presented here, using the same classification scheme as ref. ^35^.

### Downhole measurements and core-log-seismic integration

Two downhole-logging runs were carried out in Hole U1523D using a modified triple combo and FMS-sonic tool strings^35^. Down-hole acoustic porosity was derived indirectly from the sonic log (Sonic derived Porosity, SPHI^89^). Vp/Vs was derived from the ratio of compressional to shear wave velocity. The log curves were depth matched using the total gamma-ray log from the triple combo tool string and depth-shifted to the seafloor^35^. The data were processed at Lamond Doherty Earth Observatory following standardised procedures, including removing depth offsets between logging runs, data conversion and image creation^35^. Data were visualised using Schlumberger Petrel and Techlog software. Discrepancies in the extent of individual tool measurements downhole are due to different tool positions along the ~50 m-long tool string^35^.

Downhole-log data were combined with core analysis from Holes U1523A, B and E and seismic-reflection data. Lithostratigraphic and core measurement depths were converted from depth in metres to two-way-travel time to create a depth-travel time relationship using the Petrel software package, using P-wave calliper point-source velocity data from physical property measurements and in-situ and continuous sonic velocity data from the Dipole Sonic Imager collected during downhole-logging operations^35^.

### Chronology

A revised chronology was developed for IODP Site U1523 (Supplementary Fig. 8) based on diatom, radiolarian, and marine palynomorph (dinocyst) biostratigraphic events presented in Supplementary Table 1. The revised chronology benefits from the addition of diatom analyses at 20–40 cm sample spacing, which resulted in the addition of diatom events, and adjustments to the depth of biostratigraphic events. Radiolarian and dinocyst events are from shipboard results^35^, and two magnetic polarity reversals^35^ were also identified shipboard (Supplementary Table 1).

A series of four holes at Site U1523 resulted in the development of a composite section^87^. Core recovery in each hole is presented in metres in Supplementary Fig. 8, relative to core depth below seafloor (CSF-A)^35^. First Appearance Datum (FAD) and Last Appearance Datum (LAD) events are plotted on Supplementary Fig. 8 as arrows pointing to the left or right, respectively, which help constrain an interpreted age-depth line of correlation that accommodates the biostratigraphic constraints and the two magnetic polarity events. Vertical lines reflect uncertainty in location of the biostratigraphic event due to wide sample spacing from shipboard samples or due to gaps in core recovery. The ages assigned to biostratigraphic events are derived from the Proceedings of the IODP Expedition 374 volume^35^.

Two segments of a linear age-depth line of correlation are presented in Supplementary Fig. 8 which represent a preferred interpretation for the composite of holes from Site U1523 to accommodate the available data. The upper interval between 0 and 21.70 m CSF-A is interpreted to represent the time interval from 0 to 1.78 Ma, with an average sediment accumulation rate of 1.219 cm kyr^−1^ (~72% core recovery). The lower interval between 21.70 and 48.52 m CSF-A is interpreted to represent the time interval from 1.78 Ma to 2.58 Ma, with an average sediment accumulation rate of 3.352 cm kyr^−1^ (~52% core recovery).

Most of the samples examined for diatom biostratigraphy contain poorly preserved diatoms with an abundance of inferred and obvious reworking. Of note are two diatom-rich intervals with excellent preservation at 61 and 62.1 m CSF-A. In selecting the depth of FAD and LAD events, preference was given to the biostratigraphic information derived from the discrete, rare, and discontinuous samples that yielded rich and diverse diatom assemblages. These were interpreted to result from primary sedimentation from surface waters to the seafloor, with only minimal modification by glacial and transport (reworking) processes (e.g. no fragments, pristine tests, etc.). These rich diatom-rich samples are inferred to reflect seasonally open marine conditions above Site U1523.

The coincident occurrence of numerous biostratigraphic LAD and FAD events at the boundary between Lithological Units II and III indicates the presence of a disconformity spanning the time interval from 8.2 to 4.1 Ma. The position and slope of the segment of the age-depth correlation in Unit III is transferred from that of the shipboard age model^35^.

### Weak layer and submarine landslide chronology

The minimum weak layer ages are based on core-seismic correlation by tracing the horizons directly beneath the submarine landslide scarps into the undisturbed sedimentary sequence to Site U1523. Where an age range is presented, this represents the minimum-maximum weak layer age range, based on dating constraints (Fig. 9; Supplementary Fig. 8)^35^. The minimum submarine landslide ages are based on the ages of the post-failure reflectors (hemipelagic sediments that immediately overlie the submarine landslide scarps:^20^ S1^Top^, S1b^Top^ and S2^Top^) traced into the undisturbed sedimentary sequence to Site U1523. The U1523 age model, alongside the published record from ANDRILL^53,60^, were used to constrain the weak layer and submarine landslide ages.

Chronologic uncertainty intervals for the weak layers and minimum submarine landslide ages are shown on the U1523 age model as vertical lines highlighting uncertainty (Fig. 9; Supplementary Fig. 8). Uncertainty occurs because biostratigraphic and magnetic polarity datums were used to calculate ages, which are affected by sample spacing, core gaps and fossil reworking^35^. Uncertainty is also introduced during core-seismic integration due to seismic resolution (vertical resolution is 3 m) and low sediment deposition rates (e.g. 1.219–3.352 cm kyr^−1^; Fig. 9). Uncertainties due to core-seismic integration are calculated by the limit of seismic resolution/sedimentation rate^20^. We use the U1523 age model tie points closest to each of the submarine landside scarps to calculate the rate of sediment deposition of the overlying deposits (Fig. 9).

## Supplementary information

Supplementary Material

## Data Availability

IBCSO v2 bathymetric data is available via the Pangaea library: 10.1594/PANGAEA.937574. Seismic-reflection profiles used in this study are provided in Supplementary Figs. 3–7 and are available through the Antarctic Seismic Data Library System (SDLS): https://sdls.ogs.trieste.it/cache/index.jsp. Figs. 5–8 have accessible data that can be obtained in raw format from the IODP LIMS database: https://web.iodp.tamu.edu/LORE/. The grainsize data generated in this study has been deposited in the PANGAEA database: PDI-34376. The datum points of biostratigraphic events used in the revised age model are provided in Supplementary Table 1.
