# Supplementary material for: Climate-controlled submarine landslides on the Antarctic continental margin
Source: Nat Commun. 2023 May 18;14:2714. doi: 10.1038/s41467-023-38240-y (PMC10195823; doi:10.1038/s41467-023-38240-y)
Supplement: Supplementary file 1 — Supplementary Material [file 41467_2023_38240_MOESM1_ESM.docx]

**Supplementary Material**

**Climate-controlled submarine landslides on the Antarctic continental margin**

Gales, J. A^a*^., McKay, R. M^b^., De Santis, L^c^., Rebesco, M^c^., Laberg, J. S^d^., Shevenell, A. E^e^., Harwood, D^f^., Leckie, R. M^g^., Kulhanek, D. K^h,i^., King, M^a^., Patterson, M^i^., Lucchi, R. G^c,d^., Kim, S^j^., Kim, S^k^., Dodd, J^l^., Seidenstein, J^g,m^., Prunella, C.^e,n^., Ferrante, G. M^c^. & IODP Expedition 374 Scientists.

a) School of Biological and Marine Sciences, University of Plymouth, Plymouth, UK.

b) Antarctic Research Centre, Victoria University of Wellington, Wellington, New Zealand.

c) National Institute of Oceanography and Applied Geophysics – OGS, Trieste, Italy.

d) Department of Geosciences, UIT – The Arctic University of Norway, Tromsø, Norway.

e) College of Marine Sciences, University of South Florida, St Petersburg, USA.

f) Earth and Atmospheric Sciences, University of Nebraska, Lincoln, USA.

g) Department of Earth, Geographic, and Climate Science, University of Massachusetts, Amherst, USA.

h) Institute of Geosciences, Christian-Albrechts-University of Kiel, Kiel, Germany​​​​​​​.

i) Department of Earth Sciences, Binghamton University, State University of New York, Binghamton, NY, USA.

j) Ocean Climate Response & Ecosystem Research Department, Korea Institute of Ocean Science and Technology, Busan, Republic of Korea.

k) Division of Glacial Environment Research, Korea Polar Research Institute, Incheon, Republic of Korea.

l) Department of Earth, Atmosphere and Environment, Northern Illinois University, USA.

m) Florence Bascom Geoscience Center, U.S. Geological Survey, National Center, Reston, VA, USA.

n) National Science Foundation, Alexandria, VA, USA.

*Corresponding author; [jenny.gales@plymouth.ac.uk](mailto:jenny.gales@plymouth.ac.uk)


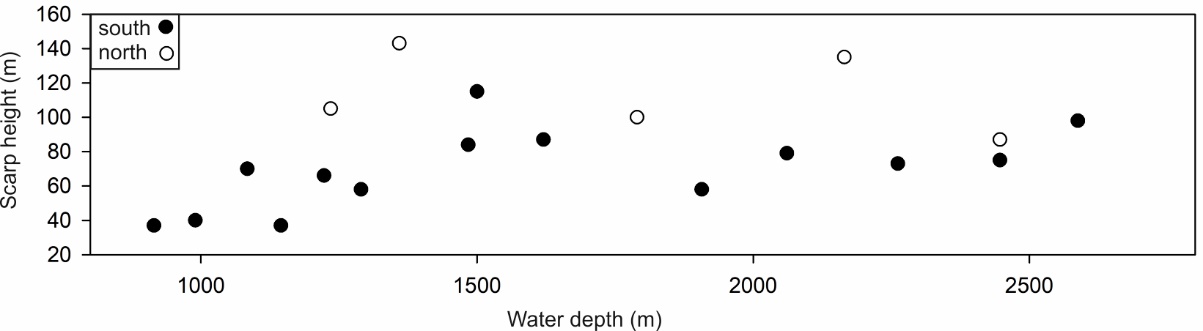


**Supplementary Figure 1.** Scarp height vs water depth of scarps in southern submarine landslide region (black) and northern submarine landslide region (white). Each point represents the average value along traceable scarp length (see Fig.2b for area of coverage).


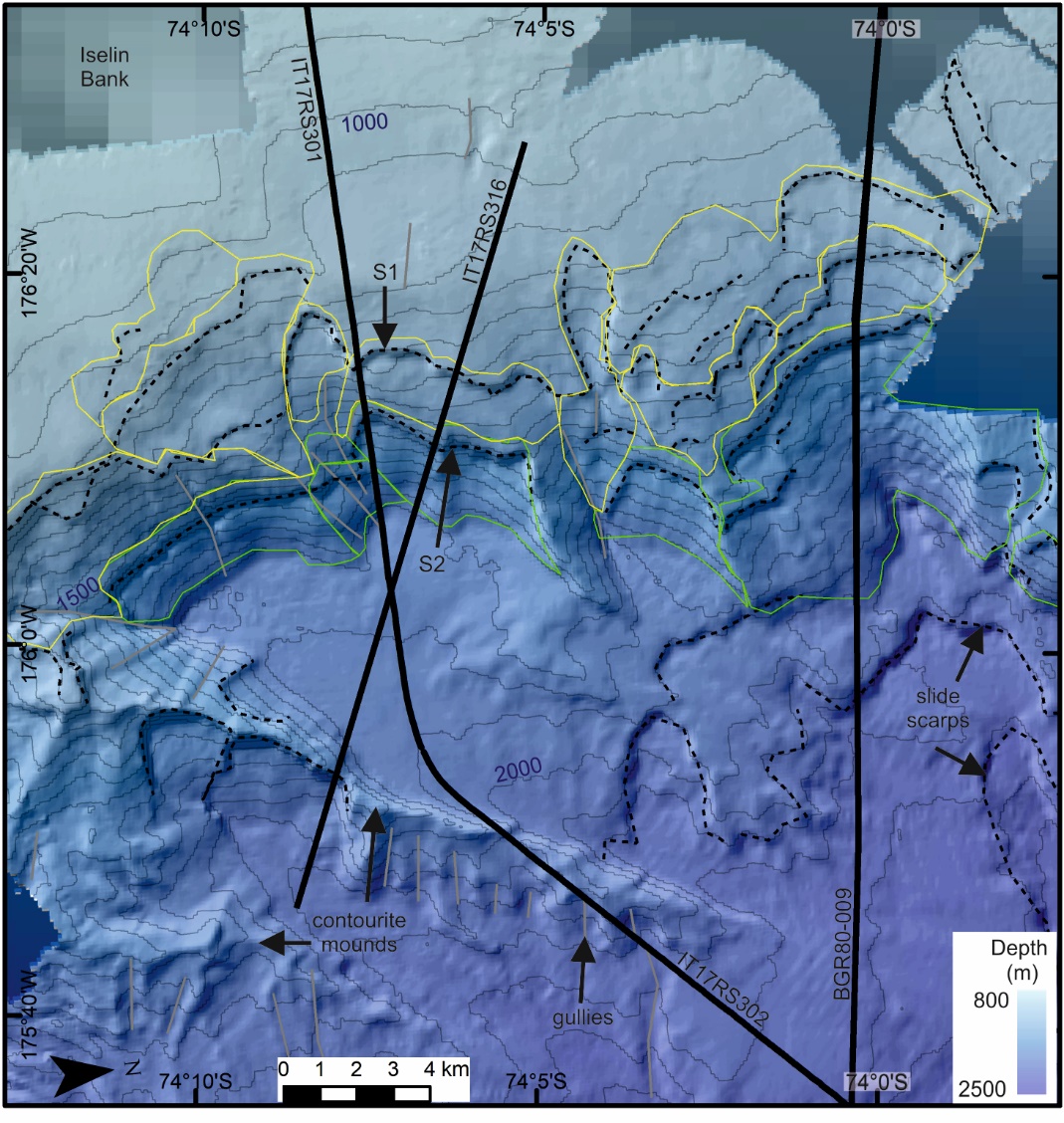


**Supplementary Figure 2.** Polygons used for submarine landslide volume calculations in the southern submarine landslide region. S1 is shown by yellow lines; S2 is shown by green lines. Hillshaded multibeam echosounder data gridded at 30-m cell size overlying IBCSO v2 regional bathymetry^90^. Contours are spaced at 50 m and labelled every 500 m. Black dashed lines are location of submarine landslide scarps on the slope and rise. Black solid lines show locations of seismic lines IT17RS301, IT17RS302, IT17RS316 and BGR80-009.

**
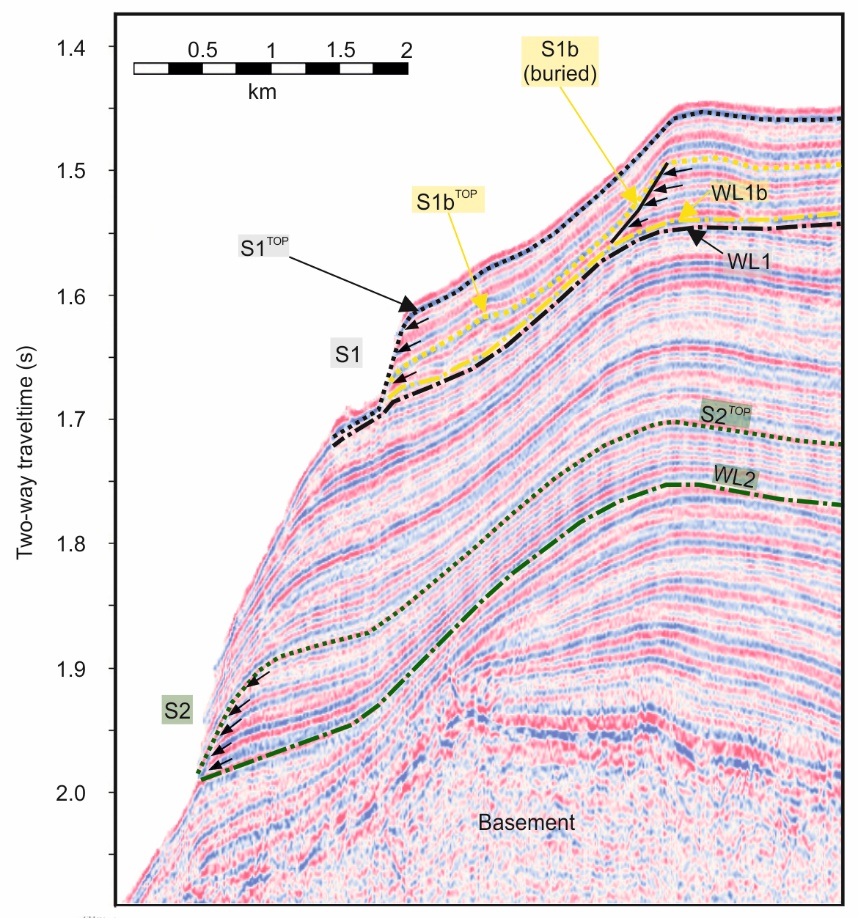
**

**Supplementary Figure 3.** Interpreted seismic reflection profile of the Iselin Bank shelf edge showing three submarine landslide scarps (S1, S1b and S2) and associated weak layers (dash-dotted lines with labels WL1, WL1b, and WL2). Location is shown in Fig.3. Seismic line is IT17RS301. Dotted lines show correlation lines used to calculate minimum submarine landslide ages by locating packages immediately overlying associated weak layers S1^Top^, S1b^Top^ and S2^Top^. Small black arrows show the termination of internal reflectors against scarps. Vertical exaggeration is 12.


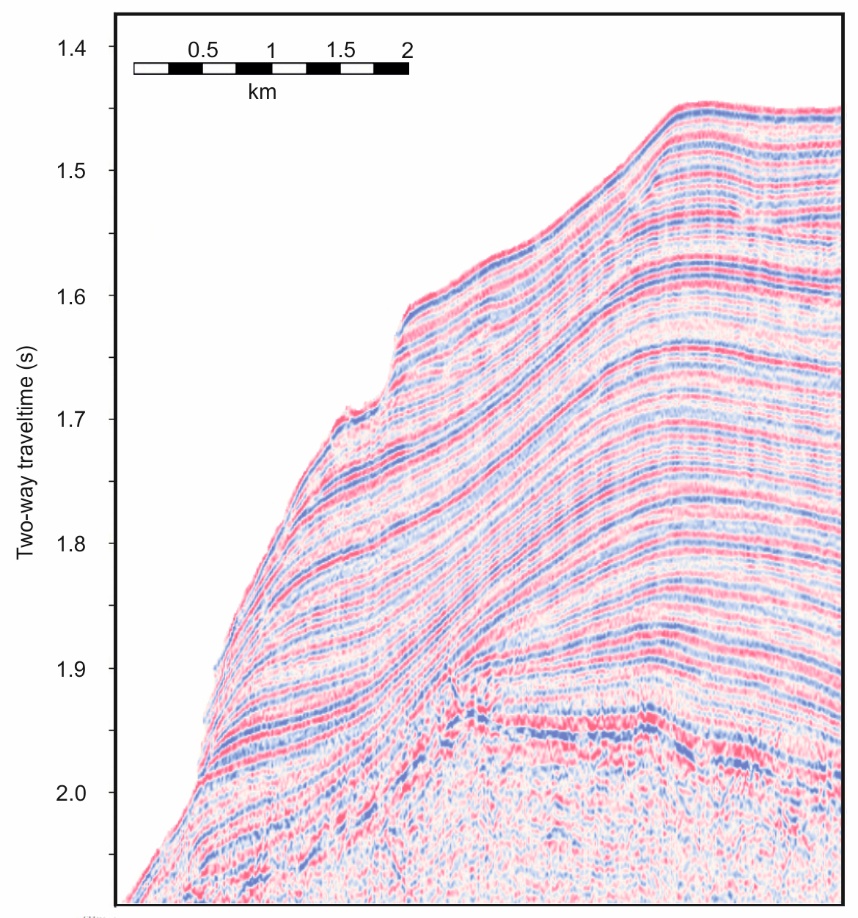


**Supplementary Figure 4.** Uninterpreted seismic reflection profile from line IT17RS301. Vertical exaggeration is 12.


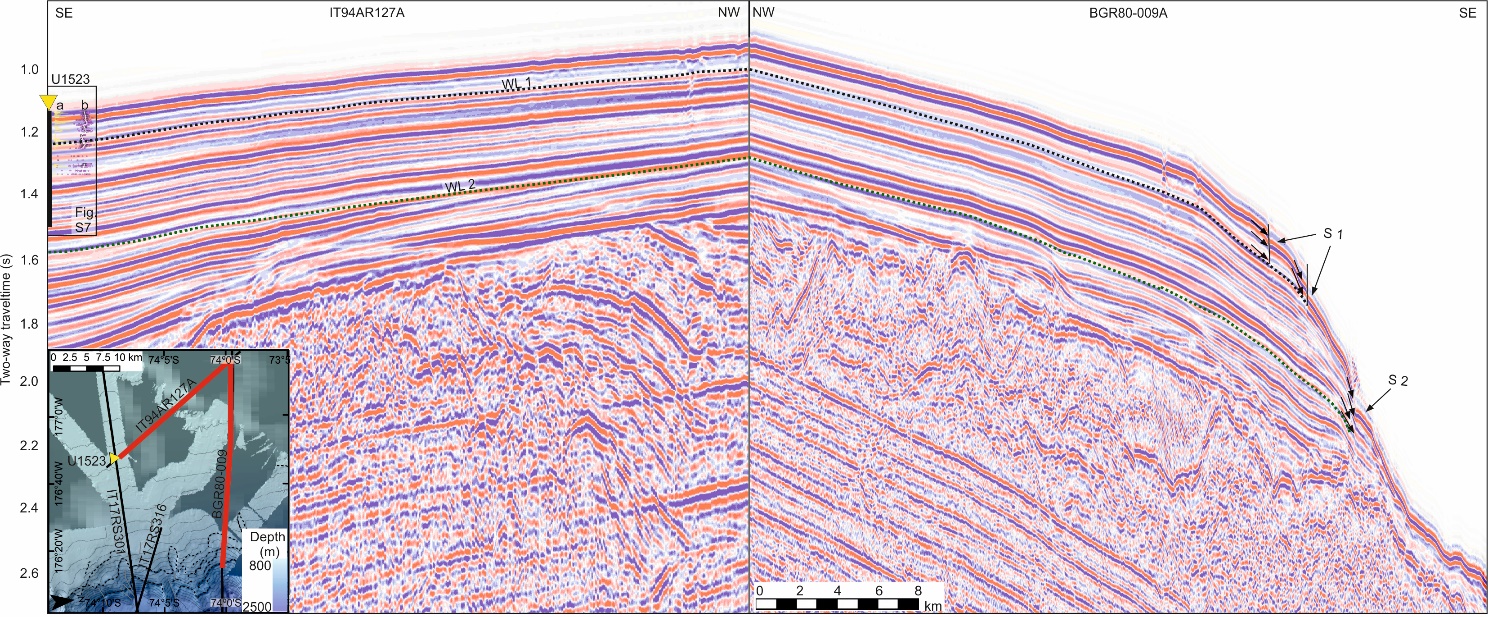


**Supplementary Figure 5.**  Interpreted seismic reflection profile integrated with core-logs from IODP Site U1523 on the Iselin Bank. **A.** Seismic lines are IT94AR127A (section) and BGR80-009A (section). Yellow triangle locates IODP Site U1523 with drilled core extent shown by black vertical line. Selected core physical properties logs are shown alongside core extent: (a) P-wave velocity and (b) gamma ray attenuation. For detail, see Fig.6 and Supplementary Fig.7 (black box locates Supplementary Fig.7). Submarine landslide scarps are labelled S1 and S2. Small black arrows show the termination of internal reflectors against scarps. Weak layers (WL1 and WL2) are located by grey dashed line (WL1) and green dashed line (WL2). Vertical exaggeration is 8. Inset figure locates seismic sections shown above.


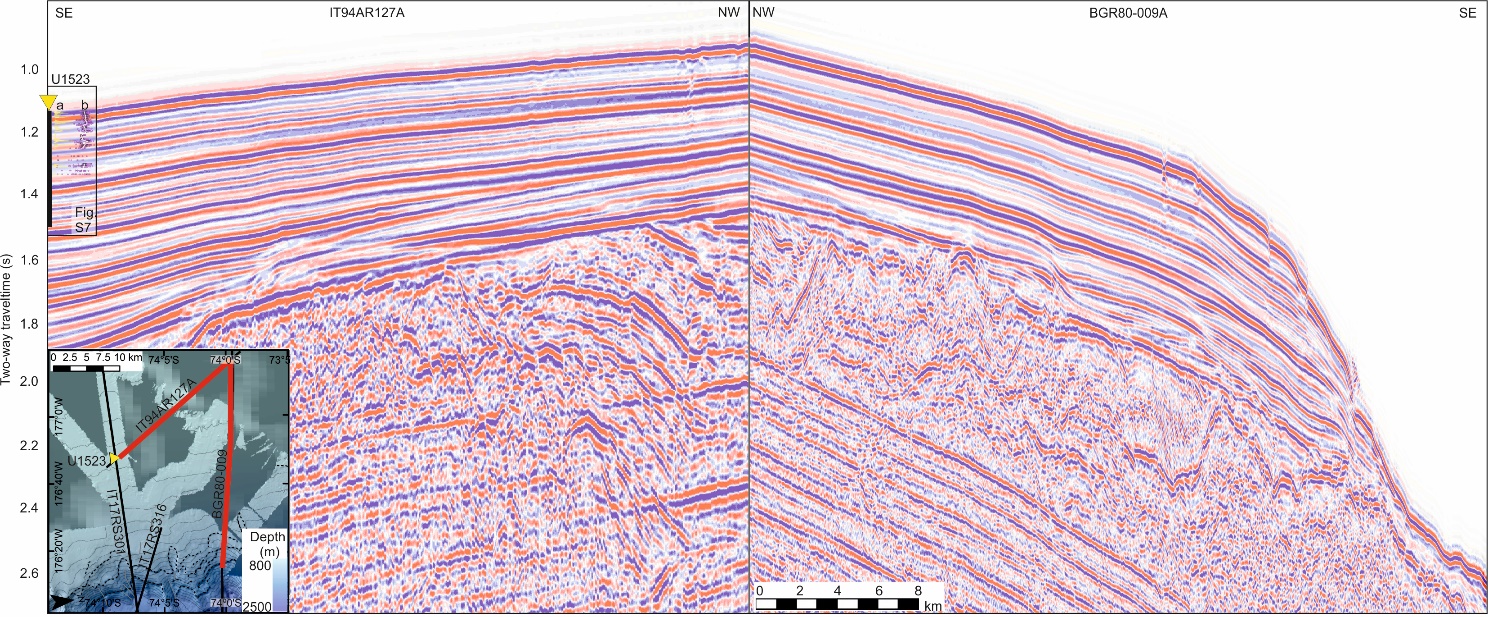


**Supplementary Figure 6.** Uninterpreted seismic reflection profile integrated with core-logs from IODP Site U1523 on the Iselin Bank. Seismic lines IT94AR127A (section) and BGR80-009A (section). Yellow triangle locates IODP Site U1523 with drilled core extent shown by black vertical line. Selected core physical properties logs are shown alongside core extent: (a) P-wave velocity and (b) gamma ray attenuation. For detail, see Fig.6 and Supplementary Fig.7 (black box locates Supplementary Fig.7). Vertical exaggeration is 8. Inset figure locates seismic sections shown above.


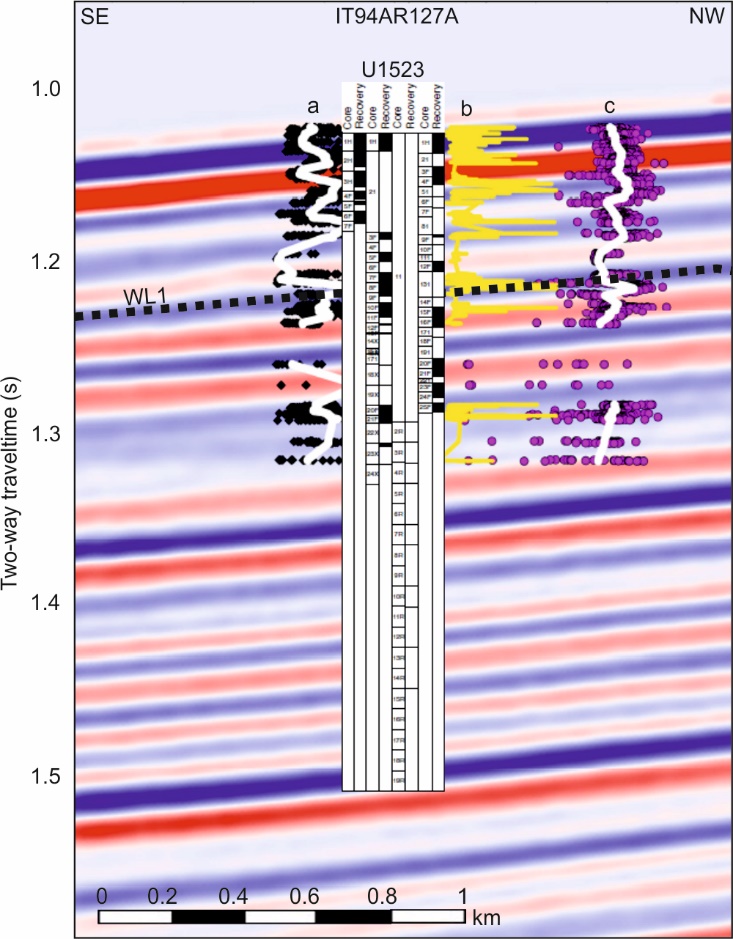


**Supplementary Figure 7.** Close up of core-log-seismic integration on the Iselin Bank (section shown in Supplementary Fig.5). Seismic line is IT94AR127A (section). Site U1523 position and core recovery is shown by black shaded intervals in overlaid core-log. Selected core physical properties logs are shown alongside core extent: (a) Magnetic Susceptibility, (b) P-wave velocity and (b) gamma ray attenuation. For detail see Fig.6.

**
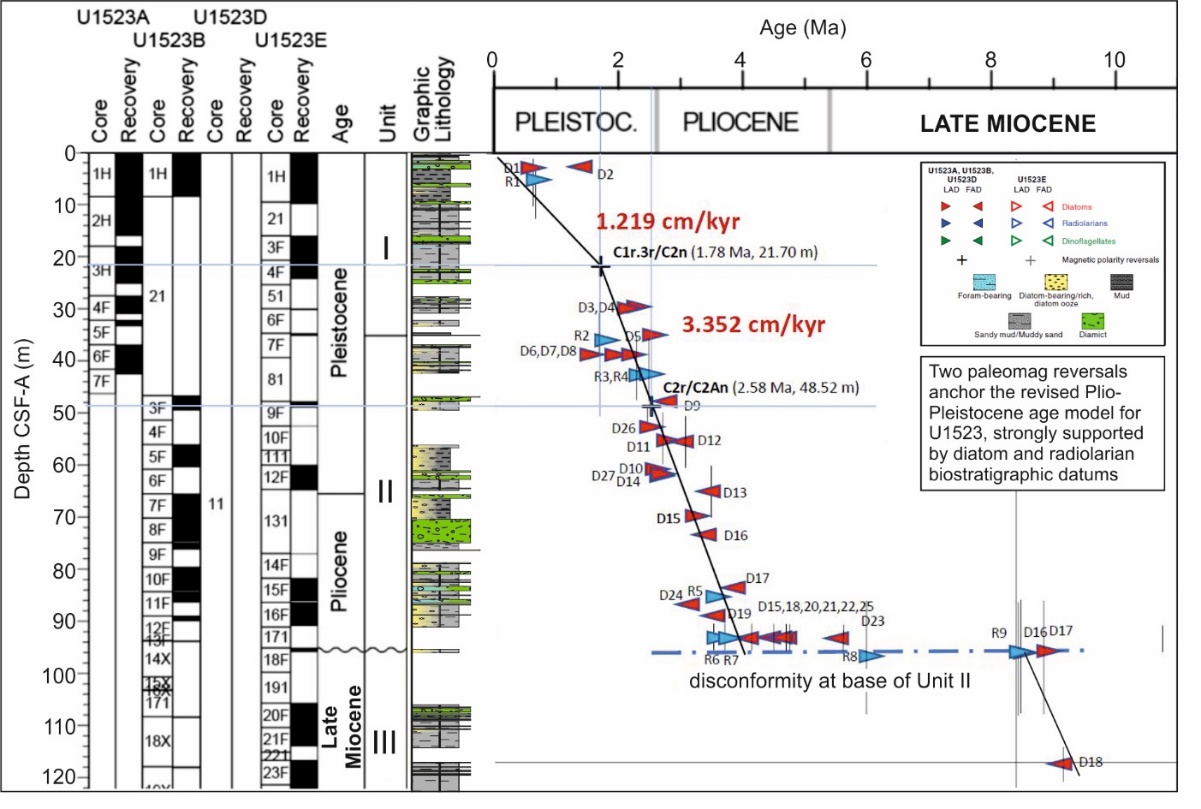
**

**Supplementary Figure 8.** Age model for IODP Site U1523 based on biostratigraphic events from diatoms, radiolarians and marine palynomorphs (dinocysts) presented in Supplementary Table 1. This revises the shipboard age model^35^. FAD is First Appearance Datum. LAD is Last Appearance Datum. Black crosses are magnetic polarity events. Vertical lines reflect uncertainty in location of the biostratigraphic event due to wide sample spacing from shipboard samples or due to coring recovery gaps. Blue dashed line is disconformity at the base of Unit II. Average sedimentation rates are shown in red.


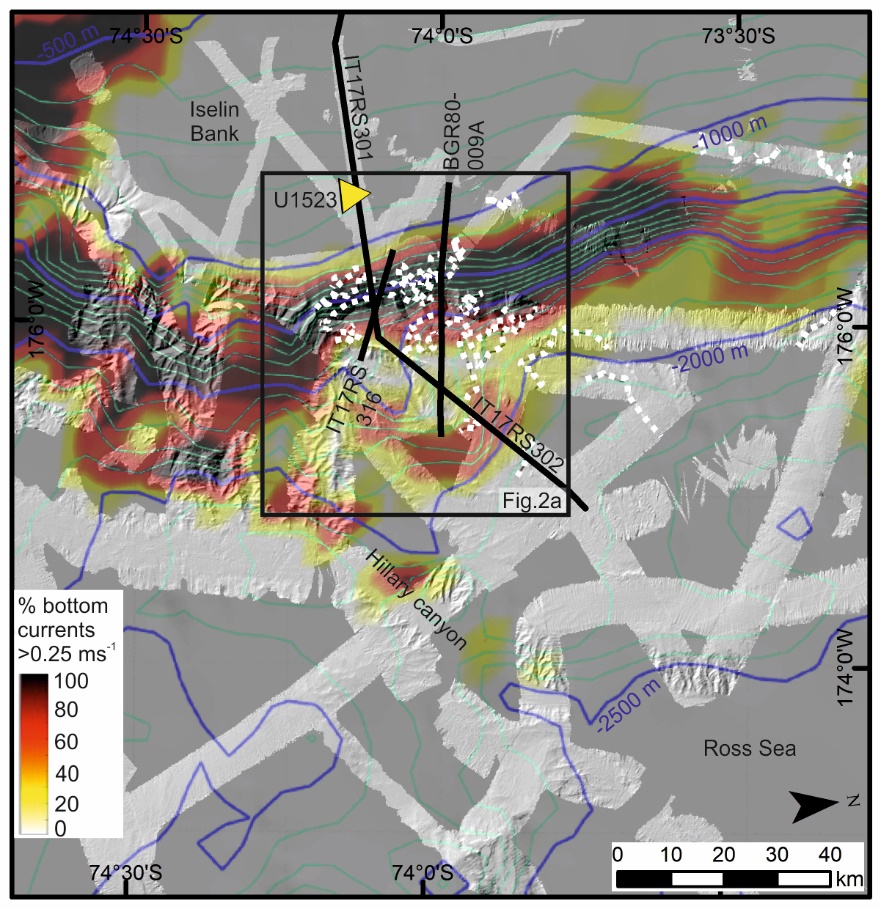


**Supplementary Figure 9.** Modelled occurrence (%) of bottom-current velocities >0.25 ms^-1^ over a 5-year period along the Iselin Bank. Modelled bottom-current velocities >0.25 ms^-1^ derived by Conte et al.^36^. Hillshaded multibeam echosounder data is gridded at 30-m cell size with contours every 100 m (green) and 500 m (blue). Yellow triangle locates IODP Site U1523. Black lines locate seismic profiles. Black box locates study area shown in Fig.2a. White dashed lines mark scarp positions.

**Supplementary Table 1.** Datum points of biostratigraphic events from diatoms, radiolarians, marine palynomorphs (dinocysts) used in revised age model for IODP Site U1523.

| **Age EXP 374** | **Code** | | **Datum type** | **change?** | | **species/event name** | **Top Depth (m CSF-A)** | **Base Depth (m CSF-A)** | **Hole** | **Sample** |
| --- | --- | --- | --- | --- | --- | --- | --- | --- | --- | --- |
| 0.65 | R1 | | LAD | nc | | *Antarctissa cylindrica* | 0.00 | 9.81 | E | 1H-CC |
| 0.60 | D1 | | LAD | new | | *Actinocyclus ingens* |  |  | A | 1-2, 62-62 cm |
| 1.40 | D2 | | FAD | new | | *Fragilariopsis separanda* |  |  | A | 1-2, 62-62 cm |
| 1.78 | Pol | |  |  | | C1r.3r/2n |  | 21.70 | E | 4F-1, 100-101 cm |
| 2.10 | D3 | | LAD | shift | | *Actinocyclus fasciculatus* |  |  | A | 4-2, 122-123 cm |
| 2.20 | D4 | | LAD | new | | *Denticulopsis maccolumii* |  |  | A | 4-2, 122-123 cm |
| 2.40 | D5 | | LAD | new | | *Rouxia diploneides* |  |  | E | 7-CC, 6-7 cm |
| 1.50 | D6 | | LAD | new | | *Rouxia antarctica* |  |  | A | 6-2, 22-23 cm |
| 1.90 | D7 | | LAD | shift | | *Thalassiosira kolbei* |  |  | A | 6-2, 22-23 cm |
| 2.20 | D8 | | LAD | new | | *Thalassiosira vulnifica* |  |  | A | 6-2, 22-23 cm |
| 2.75 | D9 | | FAD | new | | *Actinocyclus actinochilus* |  |  | B | 3-1, 69-70 cm |
| 2.50 | D10 | | LAD | shift | | *Thalassiosira complicata* |  |  | E | 12-1, 99-100 cm |
| 1.72 | R2 | | LAD | nc | | *Cycladophora pliocenica* | 30.98 | 41.59 | A | 6F-CC (ship) |
| 2.35 | R3 | | LAD | nc | | *Helotholus vema* | 35.10 | 48.77 | E | 9F-1, 0-5 cm |
| 2.58 | Pol | |  |  | | C2r/C2An |  | 48.52 | B | 3F-2, 40-41 cm |
| 2.47 | R4 | | LAD | nc | | *Desmospyris spongiosa* | 35.10 | 48.77 | E | 9F-1, 0-5 cm |
| 2.70 | D11 | | LAD | new | | *Fragilariopsis weaverii* | 49.50 | 60.32 | B | 5F-CC |
| 3.10 | D12 | | LAD | new | | *Fragilariopsis tigris* | 49.50 | 60.32 | B | 5F-CC |
| 3.50 | D13 | | FAD | new | | *Fragilariopsis weaverii* | 60.32 | 60.32 + ~10 | B | 5F-CC |
| 2.60 | D14 | | LAD | new | | *Thalassiosira insigna* |  |  | E | 12-2, 19-20 cm |
| 3.20 | D15 | | FAD | new | | *Fragilariopsis tigris* |  |  | B | 7-CC |
| 3.40 | D16 | | FAD | new | | *Thalassiosira insigna* |  |  | B | 8-3, 9-109 cm |
| 3.90 | D17 | | FAD | new | | *Fragilariopsis interfrigidaria* |  |  | B | 10-3, 9-89 cm |
| 4.40 | D18 | | FAD | new | | *Fragilariopsis barronii* | 90.77 | 95.80 | E | 16-CC |
| 3.60 | D19 | | FAD | new | | *Rhizosolenia harwoodii* |  |  | E | 16-3, 109-110 cm |
| 2.70 | D27 | | FAD | shift | | *Actinocyclus fasciculatus* |  |  | E | 12-1, 59-60 cm |
| 2.50 | D26 | | LAD |  | | *Rouxia diploneides* | 48.77 | 52.50 | E | 10-CC |
| 3.20 | D24 | | FAD |  | | *Thalassiosira vulnifica* | 86.30 | 108.47 | B | 11F-CC |
| 4.10 | D20 | | FAD |  | | *Thalassiosira kolbei* | 90.77 | 95.80 | E | 16-CC |
| 4.60 | D21 | | FAD |  | | *Rouxia diploneides* | 90.77 | 95.80 | E | 16-CC |
| 4.70 | D22 | | FAD |  | | *Thalassiosira complicata* | 90.77 | 95.80 | E | 16-CC |
| 4.50 | D25 | | FAD |  | | *Thalassiosira striata* | 90.77 | 95.80 | E | 16-CC |
| 5.60 | D23 | | FAD |  | | *Thalassiosira inura* | 90.77 | 95.80 | E | 16-CC |
| 3.53 | R6 | | LAD | nc | | *Prunopyle titan ^* | 90.77 | 95.80 | E | 18X-CC |
| 3.72 | R7 | | LAD | nc | | *Lampromitra coronata* | 90.77 | 95.80 | E | 18F-CC |
| 5.95 | R8 | | LAD | nc | | *Desmospyris rhodospyroides ^* | 86.38 | 108.47 | B | 18X-CC |
| 8.35 | R9 | | LAD | nc | | *Prunopyle hayesi ^* | 86.38 | 108.47 | B | 18X-CC |
| 8.40 | D16 | | LAD | nc | | *Denticulopsis ovata* | 86.38 | 108.47 | B | 18X-CC |
| 8.80 | D17 | | LAD | nc | | *Fragilariopsis januaria* | 86.38 | 108.47 | B | 18X-CC |
| 9.10 | D18 | | FAD | nc | | *Thalassiosira torokina* | 113.95 | 121.45 | E | 21F-CC |
| < 8.60 | | R10 | FAD (-) | |  | *Antarctissa strelkovi ¯* | 110.40 | 113.95 | E | 20F-CC |
| 8.42 | R11 | | LAD | nc | | *Acrosphaera australis ^* | 86.38 | 108.47 | B | 18X-CC |
| 7.30 | PL1 | | LAD | nc | | *Selenopemphix bothrion* " | 86.38 | 108.47 | B | 18X-CC |
| 7.30 | PL2 | | LAD | nc | | *Selenopemphix minys "* | 86.38 | 108.47 | B | 18X-CC |
| 8.62 | R12 | | LAD | nc | | *Cycladophora spongothorax ^* | 86.38 | 108.47 | B | 18X-CC |

**Supplementary Material 10.** IODP Expedition 374 Scientists.

Jeanine Ash^a^, François Beny^b^, Imogen M. Browne^c^, Giuseppe Cortese^d^, Laura De Santis^e^, Justin P. Dodd^f^, Oliver M. Esper^g^, Jenny A. Gales^h^, David M. Harwood^i^, Saki Ishino^j^, Benjamin A. Keisling^k^, Sookwan Kim^l^, Sunghan Kim^m^, Denise K. Kulhanek^n,q^, Jan Sverre Laberg^o^, R. Mark Leckie^k^, Robert M. McKay^p^, Juliane Müller^g^, Molly O. Patterson^q^, Brian W. Romans^r^, Oscar E. Romero^s^, Francesca Sangiorgi^t^, Osamu Seki^u^, Amelia E. Shevenell^c^, Shiv M. Singh^v^, Isabela M. Cordeiro de Sousa^w^, Saiko T. Sugisaki^x^, Tina van de Flierdt^y^, Tim E. van Peer^z^, Whenshen Xiao^aa^, Zhifang Xiong^bb^.

^a^Department of Earth, Environmental and Planetary Sciences, Rice University, Houston, USA.

^b^Laboratoire d’Océanologie et de Géosciences, Université de Lille I, Villeneuve d’Ascq, France.

^c^College of Marine Science, University of South Florida, St. Petersburg, USA.

^d^GNS Science, Lower Hutt, New Zealand.

^e^Instituto Nazionale di Oceanografia e di Geofisica Sperimentale (OGS), Trieste, Italy.

^f^Department of Earth, Atmosphere and Environmental Geosciences, Northern Illinois University, USA.

^g^Alfred Wegener Institute, Bremerhaven, Germany.

^h^School of Biological & Marine Sciences, University of Plymouth, Plymouth, UK.

^i^Earth and Atmospheric Sciences, University of Nebraska, Lincoln, USA.

^j^Department of Earth and Planetary Sciences, Nagoya University, Nagoya, Japan.

^k^Department of Earth, Geographic, and Climate Science, University of Massachusetts, Amherst, USA.

^l^Ocean Climate Response & Ecosystem Research Department, Korea Institute of Ocean Science and Technology, Busan, Republic of Korea.

^m^Division of Polar Paleoenvironment, Korea Polar Research Institute, Republic of Korea.

^n^Institute of Geosciences, Christian-Albrechts-University of Kiel, Kiel, Germany​​​​​​​.

^o^Department of Geosciences, UIT – The Arctic University of Norway, Tromsø, Norway.

^p^Antarctic Research Centre, Victoria University of Wellington, Wellington, New Zealand.

^q^Department of Earth Sciences, Binghamton University, State University of New York, Binghamton, USA.

^r^Geosciences, Virginia Tech, Blacksburg, USA.

^s^MARUM, University of Bremen, Bremen, Germany.

^t^Earth Sciences, University of Utrecht, Utrecht, The Netherlands.

^u^Institute of Low Temperature Science, Hokkaido University, Sapporo Hokkaido, Japan.

^v^Polar Biology Lab, National Centre for Antarctic and Ocean Research (NCAOR), Goa, India.

^w^Instituto de Geociencias, Universidade de Brasília, Brasilia, Brazil.

^x^Marine Geology Research Group, Geological Survey of Japan, Tsukuba Ibaraki, Japan.

^y^Department of Earth Science & Engineering, Imperial College London, London, UK.

^z^National Oceanography Centre Southampton, University of Southampton, Southampton, UK.

^aa^State Key Laboratory of Marine Geology, Tongji University, Shanghai, China.

^bb^First Institute of Oceanography, State Oceanic Administration, Qingdao, China.
